# A Large Deviation Principle in Many-Body Quantum Dynamics

**DOI:** 10.1007/s00023-021-01044-1

**Published:** 2021-04-08

**Authors:** Kay Kirkpatrick, Simone Rademacher, Benjamin Schlein

**Affiliations:** 1 Department of Mathematics, University of Illinois at Urbana-Champaign, Urbana, IL 61801 USA; 2 Institute of Science and Technology Austria, Am Campus 1, 3400 Klosterneuburg, Austria; 3 Institute of Mathematics, University of Zurich, Winterthurerstrasse 190, 8057 Zurich, Switzerland

## Abstract

We consider the many-body quantum evolution of a factorized initial data, in the mean-field regime. We show that fluctuations around the limiting Hartree dynamics satisfy large deviation estimates that are consistent with central limit theorems that have been established in the last years.

## Introduction

A system of *N* bosons in the mean-field regime can be described by the Hamilton operator

$$\begin{aligned} H_N = \sum _{j=1}^N -\Delta _{x_j} + \frac{1}{N} \sum _{i < j}^N v (x_i - x_j) \end{aligned}$$

acting on the Hilbert space $L^2_s ({\mathbb {R}}^{3N})$, the subspace of $L^2 ({\mathbb {R}}^{3N})$ consisting of functions that are symmetric with respect to any permutation of the *N* particles.

The time evolution of the *N* particles is governed by the many-body Schrödinger equation

1.1 $$\begin{aligned} i\partial _t \psi _{N,t} = H_N \psi _{N,t} \; . \end{aligned}$$

If the *N* particles are trapped into a finite region by a confining external potential $v_\text {ext}$, the system exhibits, at zero temperature, complete Bose–Einstein condensation in the minimizer of the Hartree energy functional

$$\begin{aligned} {\mathcal {E}}_\text {Hartree} (\varphi ) = \int \left[ |\nabla \varphi |^2 + v_\text {ext} |\varphi |^2 \right] \mathrm{d}x + \frac{1}{2} \int v (x-y) |\varphi (x)|^2 |\varphi (y)|^2 \mathrm{d}x \mathrm{d}y \end{aligned}$$

taken over $\varphi \in L^2 ({\mathbb {R}}^3)$ with $\Vert \varphi \Vert = 1$. For this reason, from the point of view of physics, it is interesting to study the solution of (1.1) for an initial sequence $\psi _N \in L^2_s ({\mathbb {R}}^{3N})$ exhibiting complete Bose–Einstein condensation, in the sense that the one-particle reduced density $\gamma _N = \mathrm {tr}_{2, \dots , N} |\psi _N \rangle \langle \psi _N |$ associated with $\psi _N$ satisfies $\gamma _N \rightarrow |\varphi \rangle \langle \varphi |$ for a normalized one-particle orbital $\varphi \in L^2 ({\mathbb {R}}^3)$, in the limit $N \rightarrow \infty$.

To keep our analysis as simple as possible, we consider solutions of (1.1) for factorized initial data $\psi _{N,0} = \varphi ^{\otimes N}$ (which obviously exhibits condensation, since $\gamma _N = |\varphi \rangle \langle \varphi |$). Notice, however, that our approach could be extended to physically more interesting initial data exhibiting condensation.

Under quite general assumptions on the interaction potential *v*, one can show that (in contrast with factorization) the property of Bose–Einstein condensation is preserved by the many-body evolution (1.1) and that, for every fixed $t \in {\mathbb {R}}$, the reduced one-particle density $\gamma _{N,t} = \mathrm {tr}_{2, \dots , N} |\psi _{N,t} \rangle \langle \psi _{N,t}|$ is such that $\gamma _{N,t} \rightarrow |\varphi _t \rangle \langle \varphi _t|$, as $N \rightarrow \infty$. Here, $\varphi _t$ is the solution of the nonlinear Hartree equation

1.2 $$\begin{aligned} i \partial _t \varphi _t = -\Delta \varphi _t + (v * |\varphi _t|^2) \varphi _t \end{aligned}$$

with the initial data $\varphi _{t=0} = \varphi$. See for example [1, 2, 4, 10–15, 18, 24, 25].

The convergence $\gamma _{N,t} \rightarrow |\varphi _t \rangle \langle \varphi _t|$ of the reduced one-particle density associated with the solution of the Schrödinger equation (1.1) can be interpreted as a law of large numbers. For a self-adjoint operator *O* on $L^2 ({\mathbb {R}}^3)$, let $O^{(j)} = 1 \otimes \dots \otimes O \otimes \dots \otimes 1$ denote the operator on $L^2 ({\mathbb {R}}^{3N})$ acting as *O* on the *j*-th particle and as the identity on the other $(N-1)$ particles. The probability that, in the state described by the wave function $\psi \in L^2_s ({\mathbb {R}}^{3N})$, the observable $O^{(j)}$ takes values in a set $A \subset {\mathbb {R}}$ is determined by

$$\begin{aligned} {\mathbb {P}}_\psi (O^{(j)} \in A) = \big \langle \psi , \chi _A (O^{(j)}) \psi \big \rangle \, . \end{aligned}$$

For factorized wave functions $\psi _N = \varphi ^{\otimes N}$, the operators $O^{(j)}$, $j=1, \dots , N$, define independent and identically distributed random variables with average $\langle \varphi , O \varphi \rangle$. The standard law of large numbers implies that

$$\begin{aligned} \lim _{N \rightarrow \infty } {\mathbb {P}}_{\varphi ^{\otimes N}} \left( \left| \frac{1}{N} \sum _{j=1}^N O^{(j)} - \langle \varphi , O \varphi \rangle \right| > \delta \right) = 0 \end{aligned}$$

for all $\delta > 0$. The solution $\psi _{N,t}$ of the Schrödinger equation (1.1), with factorized initial data $\psi _{N,0} = \varphi ^{\otimes N}$, is not factorized. Nevertheless, the convergence of the reduced density $\gamma _{N,t} \rightarrow |\varphi _t \rangle \langle \varphi _t|$ implies that the law of large numbers still holds true, i.e., that

1.3 $$\begin{aligned} \lim _{N \rightarrow \infty } {\mathbb {P}}_{\psi _{N,t}} \left( \left| \frac{1}{N} \sum _{j=1}^N O^{(j)} - \langle \varphi _t , O \varphi _t \rangle \right| > \delta \right) = 0 \end{aligned}$$

for all $\delta > 0$; see, for example, [3].

To go beyond (1.3) and study fluctuations around the limiting Hartree dynamics, it is useful to factor out the condensate.

To reach this goal, we define the bosonic Fock space ${\mathcal {F}}= \bigoplus _{j=0}^N L^2_{\perp \varphi _t} ({\mathbb {R}}^3)^{\otimes _s j}$. On ${\mathcal {F}}$, for any $f \in L^2 ({\mathbb {R}}^3)$, we introduce the usual creation and annihilation operators $a^*(f), a(f)$, satisfying canonical commutation relations. It will also be convenient to use operator-valued distributions $a_x^*, a_x$, for $x \in {\mathbb {R}}^3$, so that

$$\begin{aligned} a^* (f) = \int f(x) \, a_x^* \, \mathrm{d}x , \qquad a(f) = \int {\bar{f}} (x) \, a_x \, \mathrm{d}x \end{aligned}$$

In terms of $a_x^*, a_x$, we can express the number of particles operator, defined by $({\mathcal {N}}\Psi )^{(n)} = n \Psi ^{(n)}$, as

$$\begin{aligned} {\mathcal {N}}= \int \mathrm{d}x \, a_x^* a_x \end{aligned}$$

More generally, for an operator *A* on the one-particle space $L^2 ({\mathbb {R}}^3)$, its second quantization $\mathrm{d}\Gamma (A)$, defined on ${\mathcal {F}}$ so that $(\mathrm{d}\Gamma (A) \Psi )^{(n)} = \sum _{j=1}^n A_j \Psi ^{(n)}$, with $A_j = 1 \otimes \dots \otimes A \otimes \dots \otimes 1$ acting non-trivially on the *j*-th particle only, can be written as

$$\begin{aligned} \mathrm{d}\Gamma (A) = \int \mathrm{d}x \mathrm{d}y \; A(x;y) \, a_x^* a_y \end{aligned}$$

where *A*(*x*; *y*) is the integral kernel of *A* (with this notation ${\mathcal {N}}= \mathrm{d}\Gamma (1)$). More details on the formalism of second quantization applied to the dynamics of mean-field systems can be found in [5].

In order to factor out the condensate, described at time $t \in {\mathbb {R}}$, by the solution $\varphi _t$ of (1.2), we observe now that every $\psi \in L^2_s ({\mathbb {R}}^{3N})$ can be uniquely written as

$$\begin{aligned} \psi = \eta _0 \varphi _t^{\otimes N} + \eta _1 \otimes _s \varphi ^{\otimes (N-1)}_t + \dots + \eta _N \end{aligned}$$

with $\eta _j \in L^2_{\perp \varphi _t} ({\mathbb {R}}^3)^{\otimes _s j}$, where $L^2_{\perp \varphi _t} ({\mathbb {R}}^3)$ denotes the orthogonal complement in $L^2 ({\mathbb {R}}^3)$ of the condensate wave function $\varphi _t$. This remark allows us to define, for every $t \in {\mathbb {R}}$, a unitary operator

$$\begin{aligned} {\mathcal {U}}_t : L^2_s ({\mathbb {R}}^{3N}) \rightarrow {\mathcal {F}}_{\perp \varphi _t}^{\le N} = \bigoplus _{j=0}^N L^2_{\perp \varphi _t} ({\mathbb {R}}^3)^{\otimes _s j} \end{aligned}$$

by setting ${\mathcal {U}}_t \psi = \{ \eta _0, \eta _1 , \dots , \eta _N \}$. The unitary map ${\mathcal {U}}_t$, first introduced in [20], removes the condensate wave function $\varphi _t$ and allows us to focus on its orthogonal excitations. It maps the *N*-particle space $L^2_s ({\mathbb {R}}^{3N})$ into the truncated Fock space ${\mathcal {F}}_{\perp \varphi _t}^{\le N}$, constructed over the orthogonal complement of $\varphi _t$.

The map ${\mathcal {U}}_t$ can be used to define the fluctuation dynamics (mapping the orthogonal excitations of the condensate at time $t_1$ into the orthogonal excitations of the condensate at time $t_2$):

1.4 $$\begin{aligned} {\mathcal {W}}_N (t_2 ; t_1) = {\mathcal {U}}_{t_2} \mathrm{e}^{-i H_N (t_2-t_1)} {\mathcal {U}}_{t_1}^* : {\mathcal {F}}_{\perp \varphi _{t_1}}^{\le N} \rightarrow {\mathcal {F}}_{\perp \varphi _{t_2}}^{\le N} \;. \end{aligned}$$

The fluctuation dynamics satisfies the equation

$$\begin{aligned} i\partial _{t_2} {\mathcal {W}}_N (t_2 ; t_1) = {\mathcal {L}}_N (t_2) {\mathcal {W}}_N (t_2 ; t_1) \end{aligned}$$

with ${\mathcal {W}}_N (t_1;t_1) = 1$ for all $t_1 \in {\mathbb {R}}$ and with the generator ${\mathcal {L}}_N (t) = \left[ i\partial _{t} {\mathcal {U}}_{t} \right] {\mathcal {U}}_{t}^* + {\mathcal {U}}_{t} H_N {\mathcal {U}}_{t}^*$. To compute the generator ${\mathcal {L}}_N (t)$, we use the rules

1.5 $$\begin{aligned} {\mathcal {U}}_{t} a^* (\varphi _{t}) a (\varphi _{t}) {\mathcal {U}}_{t}^*= & {} N - {\mathcal {N}}_+ ({t}) , \nonumber \\ {\mathcal {U}}_{t} a^* (f) a (\varphi _{t}) {\mathcal {U}}_{t}^*= & {} a^* (f) \, \sqrt{N - {\mathcal {N}}_+ ({t})} , \nonumber \\ {\mathcal {U}}_{t} a^* (\varphi _{t}) a (f) {\mathcal {U}}_{t}^*= & {} \sqrt{N - {\mathcal {N}}_+ ({t})} \, a(f) ,\nonumber \\ {\mathcal {U}}_{t} a^* (f) a (g) {\mathcal {U}}_{t}^*= & {} a^* (f) a(g) \end{aligned}$$

for any $f,g \in L^2_{\perp \varphi _{t}} ({\mathbb {R}}^3)$. We obtain, similarly to [19], the matrix elements

1.6 $$\begin{aligned} \begin{aligned} \langle \xi _1, {\mathcal {L}}_N (t) \xi _2 \rangle&= \langle \xi _1, \mathrm{d}\Gamma (h_H (t) + K_{1,t}) \xi _2 \rangle + \text {Re} \int \mathrm{d}x\mathrm{d}y \; K_{2,t} (x;y) \, \langle \xi _1, b_x^* b_y^* \xi _2 \rangle \\&\quad - \frac{1}{2N} \langle \xi _1 , \mathrm{d}\Gamma (v *|\varphi _{t}|^2 + K_{1,t} - \mu _{t}) ({\mathcal {N}}_+ (t) - 1) \xi _2 \rangle \\&\quad + \frac{2}{\sqrt{N}} \text {Re} \, \langle \xi _1, {\mathcal {N}}_+ b ((v*|\varphi _{t}|^2) \varphi _{t}) \xi _2 \rangle \\&\quad +\frac{2}{\sqrt{N}} \int \mathrm{d}x \mathrm{d}y \; v (x-y) \text {Re} \, \varphi _{t} (x) \langle \xi _1, a_y^*a_{x'}b_{y'} \xi _2 \rangle \\&\quad + \frac{1}{2N} \int \mathrm{d}x \mathrm{d}y \,v (x-y) \langle \xi _1 , a_x^* a_y^* a_{x} a_{y} \xi _2 \rangle \, . \end{aligned} \end{aligned}$$

for any $\xi _1, \xi _2 \in {\mathcal {F}}_{\perp \varphi _{t}}^{\le N}$. Here, $h_H (t) = -\Delta + (v * |\varphi _{t}|^2)$, $K_{1,t} (x;y) = v(x-y) \varphi _{t} (x) {\overline{\varphi }}_{t} (y)$, $K_{2,t} (x;y) = v(x-y) \varphi _{t} (x) \varphi _{t} (y)$, $2\mu _{t} = \int \mathrm{d}x \mathrm{d}y \; v(x-y) \vert \varphi _{t} (x) \vert ^2 \vert \varphi _{t} (y) \vert ^2$. Moreover, we introduced the notation ${\mathcal {N}}_+ (t)$ for the number of particles operator on the space ${\mathcal {F}}_{\perp \varphi _{t}}^{\le N}$ (${\mathcal {N}}_+ (t) = \mathrm{d}\Gamma (q_{t})$, with $q_{t} = 1 - |\varphi _{t} \rangle \langle \varphi _{t}|$, if we think of ${\mathcal {F}}_{\perp \varphi _{t}}^{\le N}$ as a subspace of ${\mathcal {F}}$) and, for $f \in L^2_{\perp \varphi _{t}} ({\mathbb {R}}^3)$, we defined (using the notation introduced in [7])

1.7 $$\begin{aligned} \begin{aligned} b^* (f)&= {\mathcal {U}}_{t} \, a^* (f) \frac{a(\varphi _{t})}{\sqrt{N}} \, {\mathcal {U}}_{t}^* = a^* (f) \sqrt{1-\frac{{\mathcal {N}}_+ (t)}{N}} ,\\ b(f)&= {\mathcal {U}}_{t} \frac{a^* (\varphi _{t})}{\sqrt{N}} a(f) {\mathcal {U}}_{t}^* = \sqrt{1-\frac{{\mathcal {N}}_+ (t)}{N}} a(f) \end{aligned} \end{aligned}$$

and the corresponding operator-valued distributions $b^*_x, b_x$, for $x \in {\mathbb {R}}^3$.

In the limit of large *N*, the fluctuation dynamics ${\mathcal {W}}_N (t_2; t_1)$ can be approximated by a limiting dynamics ${\mathcal {W}}_\infty (t_2 ;t_1) : {\mathcal {F}}_{\perp \varphi _{t_1}} = \bigoplus _{j=0}^\infty L^2_{\perp \varphi _{t_1}} ({\mathbb {R}}^3)^{\otimes _s j} \rightarrow {\mathcal {F}}_{\perp \varphi _{t_2}} = \bigoplus _{j=0}^\infty L^2_{\perp \varphi _{t_2}} ({\mathbb {R}}^3)^{\otimes _s j}$ satisfying the equation

1.8 $$\begin{aligned} i\partial _t {\mathcal {W}}_\infty (t_2 ; t_1 ) = {\mathcal {L}}_\infty (t_2) {\mathcal {W}}_\infty (t_2;t_1) \end{aligned}$$

with the generator ${\mathcal {L}}_\infty (t_2)$, whose matrix elements are given by

$$\begin{aligned} \langle \xi _1 , {\mathcal {L}}_\infty (t_2) \xi _2 \rangle =&\langle \xi _1, \mathrm {d}\Gamma (h_H (t_2) + K_{1,t_2}) \xi _2 \rangle \\ {}&\quad + \frac{1}{2} \displaystyle \int \left[ K_{2,t_2} (x;y) \langle \xi _1, a_x^* a_y^* \xi _2 \rangle \right. \\ {}&\quad \quad \quad \quad \quad +\left. {\overline{K}}_{2,t_2} (x;y) \langle \xi _1, a_x a_y \xi _2 \rangle \right] \end{aligned}$$

for all $\xi _1, \xi _2 \in {\mathcal {F}}_{\perp \varphi _{t_2}}$; see [19]. (This line of research started in [17] and was further explored in [11, 16, 21]; recently, an expansion of the many-body dynamics in powers of $N^{-1}$ was obtained in [6].) Notice that ${\mathcal {L}}_\infty (t_2)$ acts on (a dense subspace of) the Fock space ${\mathcal {F}}_{\perp \varphi _{t_2}}$, constructed on the orthogonal complement of $\varphi _{t_2}$, with no restriction on the number of particles. We have the inclusions ${\mathcal {F}}_{\perp \varphi _{t_2}}^{\le N} \subset {\mathcal {F}}_{\perp \varphi _{t_2}} \subset {\mathcal {F}}= \bigoplus _{j=0}^\infty L^2 ({\mathbb {R}}^3)^{\otimes _s j}$. Observe also that ${\mathcal {L}}_\infty (t_2)$ is quadratic in creation and annihilation operators. It follows that the limiting dynamics ${\mathcal {W}}_\infty (t_2;t_1)$ acts as a time-dependent family of Bogoliubov transformations (in a slightly different setting, this was shown in [3]). In other words, introducing the notation $A (f;g)= a (f) + a^* ({\overline{g}})$ for $f \in L^2_{\perp \varphi _{t_2}} ({\mathbb {R}}^3)$ and $g \in J L^2_{\perp \varphi _{t_2}} ({\mathbb {R}}^3)$, with *J* the antilinear operator $Jf = {\overline{f}}$, we find

1.9 $$\begin{aligned} {\mathcal {W}}^*_\infty (t_2;t_1) A (f;g) {\mathcal {W}}_\infty (t_2;t_1) = A (\Theta (t_2;t_1) (f;g)) \end{aligned}$$

for a two-parameter family of operators $\Theta (t_2; t_1) : L_{\perp \varphi _{t_1}}^2 ({\mathbb {R}}^3) \oplus J L_{\perp \varphi _{t_1}}^2 ({\mathbb {R}}^3) \rightarrow L^2_{\perp \varphi _{t_2}} ({\mathbb {R}}^3) \oplus J L^2_{\perp \varphi _{t_2}} ({\mathbb {R}}^3)$.

The convergence towards the limiting Bogoliubov dynamics (1.8) has been used in [3, 8] to prove that, beyond the law of large numbers (1.3), the variables $O^{(j)}$ also satisfy the central limit theorem

1.10 $$\begin{aligned} \lim _{N\rightarrow \infty } {\mathbb {P}}_{\psi _{N,t}} \left( \frac{1}{\sqrt{N}} \sum _{j=1}^N \left( O^{(j)} - \langle \varphi _t , O \varphi _t \rangle \right) < x \right) = \frac{1}{\sqrt{2\pi }\, \alpha _t} \int _{-\infty }^x \mathrm{e}^{- r^2 / (2\alpha _t^2)} dr \nonumber \\ \end{aligned}$$

with $\alpha _t = \Vert f_{0;t} \Vert _2$. Here, $f_{s;t} \in L^2_{\perp \varphi _s} ({\mathbb {R}}^3 )$ satisfies the equation (for all $0\le s \le t$)

1.11 $$\begin{aligned} i\partial _s f_{s;t} = (h_H (s) + K_{1,s} + J K_{2,s}) f_{s;t}, \end{aligned}$$

with $f_{t;t}= q_t O \varphi _t = O \varphi _t - \langle \varphi _t , O \varphi _t \rangle \varphi _t$, $h_H (s) = -\Delta + (v * |\varphi _{s}|^2)$, $K_{1,s} (x;y) = v(x-y) \varphi _{s} (x) {\overline{\varphi }}_{s} (y)$ and $K_{2,s }(x;y) = v(x-y) \varphi _{s} (x) \varphi _{s} (y)$. (The solution of (1.11) is related with the family of Bogoliubov transformations $\Theta (t_1 ; t_2)$, since $\Theta (0;t) (f_{t; t} ;J f_{t;t} ) = (f_{0;t} ; J f_{0;t})$.)

For singular interaction potentials, scaling as $N^{3\beta } v (N^\beta x)$ for a $0< \beta < 1$ and converging therefore to a $\delta$-function as $N \rightarrow \infty$, the validity of a central limit theorem of the form (1.10) was recently established in [22]; in this case, the correlation structure produced by the interaction affects the variance of the limiting Gaussian distribution. For $\beta =1$ (the Gross–Pitaevskii regime), the validity of a central limit theorem for the ground state was established instead in [23].

In our main theorem, we show, for bounded interactions, a large deviation principle for the fluctuations of the many-body quantum evolution around the limiting Hartree dynamics.

### Theorem 1.1

Let $v \in L^1 ({\mathbb {R}}^3) \cap L^\infty ({\mathbb {R}}^3)$. Let *O* be a bounded self-adjoint operator on $L^2 ({\mathbb {R}}^3)$, with $\Vert \Delta O (1-\Delta )^{-1} \Vert _\text {op} < \infty$. Let $\varphi \in H^4 ({\mathbb {R}}^3)$, with $\Vert \varphi \Vert = 1$. For $t \in {\mathbb {R}}$, let $\psi _{N,t}$ denote the solution of the many-body Schrödinger equation (1.1), with initial data $\psi _{N,0} = \varphi ^{\otimes N}$. Then, there exists a constant $C > 0$ (depending only on $\Vert \varphi \Vert _{H^4}$) such that, denoting by $O^{(j)} = 1 \otimes \cdots \otimes O \otimes \cdots \otimes 1$ the operator *O* acting only on the *j*-th particle,

1.12 $$\begin{aligned} \frac{1}{N} \log {\mathbb {E}}_{\psi _{N,t}} \, \mathrm {e}^{\lambda \left[ \sum _{j=1}^N (O^{(j)} - \langle \varphi _t , O \varphi _t \rangle ) \right] } \le&\frac{\lambda ^2}{2} \alpha _t^2 \nonumber \\ {}&+ C \lambda ^3 {\left| \left| \left| O \right| \right| \right| }^3 \exp (C (1+ \Vert v \Vert _1 + \Vert v \Vert _\infty ) \vert t\vert )\nonumber \\ \end{aligned}$$

for all $\lambda \le {\left| \left| \left| O \right| \right| \right| }^{-1} \mathrm{e}^{-C ( \Vert v \Vert _\infty + \Vert v \Vert _1) t }$. Here, we defined

1.13 $$\begin{aligned} {\left| \left| \left| O \right| \right| \right| } = \Vert \Delta O (1-\Delta )^{-1} \Vert _\text {op} + (1+ \Vert v \Vert _\infty + \Vert v \Vert _1) \Vert O \Vert _\text {op} \end{aligned}$$

and $\alpha _t^2 = \Vert f_{0;t} \Vert _2^2$, with *f* as defined in (1.11).

### Remark

The result and its proof can be trivially extended to particles moving in *d* dimensions, for any $d \in {\mathbb {N}}\backslash \{ 0 \}$.

It follows from (1.12) that

$$\begin{aligned}&{\mathbb {P}}_{\psi _{N,t}} \Big ( N^{-1} \sum _{j=1}^N (O^{(j)} - \langle \varphi _t , O \varphi _t \rangle )> x \Big ) \\&\quad = {\mathbb {P}}_{\psi _{N,t}} \left( \mathrm{e}^{-\lambda N x} \; \mathrm{e}^{\lambda \left[ \sum _{j=1}^N \left( O^{(j)} - \langle \varphi _t , O \varphi _t \rangle \right) \right] } > 1 \right) \\&\quad \le \mathrm{e}^{-\lambda N x} \, {\mathbb {E}}_{\psi _{N,t}} \, \mathrm{e}^{\lambda \left[ \sum _{j=1}^N (O^{(j)} - \langle \varphi _t , O \varphi _t \rangle ) \right] } \end{aligned}$$

for all $0 \le \lambda \le {\left| \left| \left| O \right| \right| \right| }^{-1} \mathrm{e}^{-C ( \Vert v \Vert _\infty + \Vert v \Vert _1) t}$. Thus,

1.14 $$\begin{aligned} {\mathbb {P}}_{\psi _{N,t}} \Big ( N^{-1} \sum _{j=1}^N (O^{(j)} - \langle \varphi _t , O \varphi _t \rangle ) > x \Big ) \le \mathrm{e}^{N \gamma (x)} \end{aligned}$$

with rate function

$$\begin{aligned} \gamma (x) = \inf _\lambda \; \left[ - \lambda x + \frac{\lambda ^2}{2} \alpha _t^2 + C \lambda ^3 {\left| \left| \left| O \right| \right| \right| }^3 \exp (C (1+ \Vert v \Vert _1 + \Vert v \Vert _\infty ) t) \right] \end{aligned}$$

where the infimum is taken over all $0 \le \lambda \le {\left| \left| \left| O \right| \right| \right| }^{-1} \exp (-C ( \Vert v \Vert _\infty + \Vert v \Vert _1) t)$. For any fixed $t > 0$, the infimum is attained at

$$\begin{aligned} \lambda _x = \frac{2x}{\alpha _t^2 + \sqrt{\alpha _t^4 + 12 C x {\left| \left| \left| O \right| \right| \right| }^3 \exp (C (1+ \Vert v \Vert _1 + \Vert v \Vert _\infty ) t)}} \end{aligned}$$

if $x > 0$ is small enough (so that $\lambda _x \le {\left| \left| \left| O \right| \right| \right| }^{-1} \exp (-C ( \Vert v \Vert _\infty + \Vert v \Vert _1) t)$). This leads (again for $x > 0$ so small that $\lambda _x \le {\left| \left| \left| O \right| \right| \right| }^{-1} \exp (-C ( \Vert v \Vert _\infty + \Vert v \Vert _1) t)$) to

$$\begin{aligned}\gamma (x)= & {} - \frac{2 x^2 \sqrt{\alpha _t^4 + 12 C x {\left| \left| \left| O \right| \right| \right| }^3 \exp (C (1+ \Vert v \Vert _1 + \Vert v \Vert _\infty ) t)}}{\left[ \alpha _t^2 + \sqrt{\alpha _t^4+ 12 C x {\left| \left| \left| O \right| \right| \right| }^3 \exp (C (1+ \Vert v \Vert _1 + \Vert v \Vert _\infty ) t)} \right] ^2} \\&+ \frac{8C x^3 {\left| \left| \left| O \right| \right| \right| }^3 \exp (C (1+ \Vert v \Vert _1 + \Vert v \Vert _\infty ) t)}{\left[ \alpha _t^2 + \sqrt{\alpha _t^4+ 12 C x {\left| \left| \left| O \right| \right| \right| }^3 \exp (C (1+ \Vert v \Vert _1 + \Vert v \Vert _\infty ) t)} \right] ^3} \; . \end{aligned}$$

Notice that, in the regime $x = y / \sqrt{N}$, $N \gamma (x) \simeq -x^2/(2\alpha _t^2)$, which is consistent with the central limit theorem (1.10), obtained in [3, 8]. This shows, in particular, that the quadratic term on the r.h.s. of (1.12) is optimal.

To prove Theorem 1.1, we first write the expectation on the l.h.s. of (1.12) as

1.15 $$\begin{aligned} \begin{aligned} {\mathbb {E}}_{\psi _{N,t}} \, \mathrm{e}^{\lambda \left[ \sum _{j=1}^N (O^{(j)} - \langle \varphi _t , O \varphi _t \rangle )\right] }&= \left\langle \psi _{N,t} , \mathrm{e}^{\lambda \left[ \sum _{j=1}^N (O^{(j)} - \langle \varphi _t , O \varphi _t \rangle ) \right] } \psi _{N,t} \right\rangle \\&= \left\langle \Omega , {\mathcal {W}}^*_N (t;0) \mathrm{e}^{\lambda \mathrm{d}\Gamma (q_t \widetilde{O}_t q_t) + \lambda \sqrt{N} \phi _+ (q_t O \varphi _t )} {\mathcal {W}}_N (t;0) \Omega \right\rangle . \end{aligned} \nonumber \\\end{aligned}$$

in terms of the fluctuation dynamics introduced in (1.4). Here, we used the choice of the initial data to write

$$\begin{aligned} \psi _{N,t} = \mathrm{e}^{-i H_N t} \varphi ^{\otimes N} = \mathrm{e}^{-i H_N t} {\mathcal {U}}_{0}^* \Omega = {\mathcal {U}}_{t}^* {\mathcal {W}}_N (t;0) \Omega \, .\end{aligned}$$

Then, we applied (1.5) to conjugate $\exp (\lambda [ \sum _{j=1}^N (O^{(j)} - \langle \varphi _t, O \varphi _t \rangle ] )$ with ${\mathcal {U}}_t$. We introduced the notation $\widetilde{O}_t = O - \langle \varphi _t, O \varphi _t \rangle$.

In the next step, motivated by the bound $\pm \mathrm{d}\Gamma (q_t \widetilde{O}_t q_t) \le c \, \Vert O \Vert {\mathcal {N}}_+ (t)$, we control the r.h.s. of (1.15), by the product

$$\begin{aligned} \left\langle \Omega , {\mathcal {W}}^*_N (t;0) \mathrm{e}^{\lambda \sqrt{N} \phi _+ (q_t O \varphi _t)/2} \mathrm{e}^{c \lambda \Vert O \Vert {\mathcal {N}}_+ (t)} \mathrm{e}^{\lambda \sqrt{N} \phi _+ (q_t O \varphi _t)/2} {\mathcal {W}}_N (t;0) \Omega \right\rangle , \end{aligned}$$

up to the exponential of a cubic expression in $\lambda$, contributing only to the last term on the r.h.s. of (1.12); this is the content of Lemma 3.1. In the next step, Lemma 3.2, we replace the fluctuation dynamics ${\mathcal {W}}_N (t;0)$ by its limit ${\mathcal {W}}_\infty (t;0)$, as defined in (1.8); as in the first step, also this replacement only produces an error cubic in $\lambda$ in (1.12). Describing the action of ${\mathcal {W}}_\infty$ through the solution of (1.11), we arrive at the product

1.16 $$\begin{aligned} \left\langle \Omega , \mathrm{e}^{\lambda \sqrt{N} \phi _+ (f_{0;t})/2} \mathrm{e}^{\lambda \kappa _t {\mathcal {N}}_+ (0)} \mathrm{e}^{\lambda \sqrt{N} \phi _+ (f_{0;t})/2} \Omega \right\rangle \end{aligned}$$

In the final step, Lemma 3.3, we estimate (1.16), concluding the proof of (1.12). This step makes use of the choice of product initial data (which implies that the expectation is taken in the vacuum); at the expenses of a longer proof, we could have proven Theorem 1.1 to a larger and physically more interesting class of initial data.

## Preliminaries

To begin with, we introduce some notation and we recall some basic facts. For a given normalized $\varphi \in L^2 ({\mathbb {R}}^3)$, we consider the Hilbert space ${\mathcal {F}}_{\perp \varphi }^{\le N} = \bigoplus _{j=0}^N L^2_{\perp \varphi } ({\mathbb {R}}^3)^{\otimes _s j}$, with the number of particles operator ${\mathcal {N}}_+ = \mathrm{d}\Gamma (1-|\varphi \rangle \langle \varphi |)$. On ${\mathcal {F}}_{\perp \varphi }^{\le N}$, we define the operators $b(f), b^* (f)$ as in (1.7). We also define

$$\begin{aligned} \phi _+ (f) = b (f) + b^* (f) , \quad \phi _- (f) = -i (b(f) - b^* (f)) \; . \end{aligned}$$

For $g_1, g_2, g,h \in L^2_{\perp \varphi } ({\mathbb {R}}^3)$, we find the commutation relations

2.1 $$\begin{aligned}&[ b(g), b(h) ]= [ b^* (g), b^* (h)] = 0, \quad [b(g), b^* (h) ] = \langle g,h \rangle \left( 1 - \frac{{\mathcal {N}}_+}{N} \right) - \frac{1}{N} a^* (h) a (g) , \end{aligned}$$

2.2 $$\begin{aligned}&\quad [ \phi _+ (h) , i \phi _- (g) ] = - 2 \text {Re } \langle h, g \rangle \left( 1 - \frac{{\mathcal {N}}_+}{N} \right) + \frac{1}{N} a^* (g) a (h) + \frac{1}{N} a^* (h) a (g) , \end{aligned}$$

2.3 $$\begin{aligned}&\quad [ b(h) , a^* (g_1) a (g_2) ] = \langle h, g_1 \rangle b (g_2) , \qquad [ b^* (h) , a^* (g_1) a (g_2) ] = - \langle g_2 , h \rangle b^* (g_1) , \end{aligned}$$

2.4 $$\begin{aligned}&\quad [ \phi _+ (h) , {\mathcal {N}}_+ ] = i \phi _- (h), \quad [ i\phi _- (h), {\mathcal {N}}_+ ] = \phi _+ (h) .\end{aligned}$$

More generally,

2.5 $$\begin{aligned}{}[ \phi _+ (h) , \mathrm {d}\Gamma (H) ] = i \phi _- (Hh), \quad [ i\phi _- (h), \mathrm {d}\Gamma (H) ] = \phi _+ (Hh) \end{aligned}$$

for any self-adjoint operators *H*.

We also recall the bounds

2.6 $$\begin{aligned} \Vert b(h) \xi \Vert \le \Vert h \Vert _2 \Vert {\mathcal {N}}_+^{1/2} \xi \Vert , \qquad \Vert b^* (h) \xi \Vert \le \Vert h \Vert _2 \Vert ({\mathcal {N}}_+ + 1)^{1/2} \xi \Vert , \end{aligned}$$

valid for any $h \in L^2_{\perp \varphi } ({\mathbb {R}}^3)$ and the estimate

2.7 $$\begin{aligned} \pm \mathrm{d}\Gamma (H) \le \Vert H \Vert _\text {op} \, {\mathcal {N}}_+ \end{aligned}$$

for every bounded operator *H* on $L^2_{\perp \varphi } ({\mathbb {R}}^3)$. For more details, we refer to [7, Section 2].

Furthermore, we introduce the notation $\mathrm {ad}_{B}^{(n)} \left( A \right)$ defined for two operators *A*, *B* recursively by

$$\begin{aligned} \mathrm {ad}^{(0)}_B \left( A \right) = A, \quad \mathrm {ad}_{B}^{(n)} \left( A \right) = \left[ A, \; \mathrm {ad}_{B}^{(n-1)} \left( A \right) \right] . \end{aligned}$$

### Lemma 2.1

Let $$h,g \in L^2_{\perp \varphi } \left( {\mathbb {R}}^3 \right) $$. Then

2.8 $$\begin{aligned} \begin{aligned} \mathrm {ad}_{\sqrt{N}\phi _+(h)}^{(2n+1)}\left( b(g) \right)&= - 2^{2n} \sqrt{N} \Vert h \Vert ^{2n}_2 \langle g, h \rangle \left( 1- \frac{{\mathcal {N}}_+}{N} \right) \\&\quad +(2^{2n} -1) \frac{1}{ \sqrt{N}} \Vert h \Vert _2^{2n-2} \langle g , h \rangle a^* (h) a(h) \\&\quad + \frac{1}{\sqrt{N}} \Vert h \Vert _2^{2n} a^* (h) a (g) \end{aligned} \end{aligned}$$

for all $n \ge 0$ and

2.9 $$\begin{aligned} \mathrm {ad}_{\sqrt{N}\phi _+(h)}^{(2n)}\left( b(g) \right) =&\left( 2^{2n-1}-1\right) \Vert h \Vert ^{2n-2}_2 \langle g, h \rangle \; i \phi _-(h)\nonumber \\ {}&\quad + \Vert h \Vert ^{2n}_2 b(g) - \Vert h \Vert ^{2n-2}_2 \langle g, h \rangle \; b^* (h) \end{aligned}$$

for all $$n \ge 1$$.

### Proof

We prove the Lemma by induction. From (2.1), we find

$$\begin{aligned} \mathrm {ad}_{\sqrt{N} \phi _+(h)} \left( b(g) \right) =&[ \sqrt{N} \phi _+ (h) , b(g) ] = \sqrt{N} [b^* (h), b(g)] \\=&- \sqrt{N} \langle g,h \rangle \left( 1- \frac{{\mathcal {N}}_+}{N} \right) + \frac{1}{\sqrt{N}} a^*(h) a(g) , \end{aligned}$$

in agreement with (2.8) (for $n=0$). Now, we assume that, for a given $n \in {\mathbb {N}}$, (2.8) holds true, and we prove (2.9), with *n* replaced by $(n+1)$. To this end, we compute (using (2.8))

$$\begin{aligned} \mathrm {ad}^{(2n+2)}_{\sqrt{N} \phi _+(h)} \left( b(g) \right)&= \; [ \sqrt{N} \phi _+ (h), \mathrm {ad}^{(2n+1)}_{\sqrt{N} \phi _+(h)} \left( b(g) \right) ] \\&= \; 2^{2n} \Vert h \Vert _2^{2n} \langle g, h \rangle [ \phi _+ (h) , {\mathcal {N}}_+] \\&\quad + (2^{2n} - 1) \Vert h \Vert _2^{2n-2} \langle g ,h \rangle [ \phi _+ (h), a^* (h) a(h) ] \\&\quad + \Vert h \Vert _2^{2n} [ \phi _+(h) , a^* (h) a (g) ] . \end{aligned}$$

With (2.3) and (2.4), we obtain (using the identity $2^{2n} + (2^{2n} - 1) = 2^{2n+1} -1$)

$$\begin{aligned} \mathrm {ad}^{(2n+2)}_{\sqrt{N} \phi _+(h)} \left( b(g) \right) =&(2^{2n+1} -1) \Vert h \Vert ^{2n}_2 \langle g, h \rangle i \phi _- (h)\\ {}&\quad + \Vert h \Vert _2^{2n+2} b(g) - \Vert h \Vert _2^{2n} \langle g, h \rangle b^* (h) \end{aligned}$$

as claimed in (2.9) (with *n* replaced by $n+1$). Finally, we assume (2.9) for a given $n \in {\mathbb {N}}$, and we show that (2.8) holds true, with the same $n \in {\mathbb {N}}$. In fact, using (2.9), we get

$$\begin{aligned} \mathrm {ad}^{(2n+1)}_{\sqrt{N} \phi _+(h)} \left( b(g) \right)&= [ \sqrt{N} \phi _+ (h), \mathrm {ad}^{(2n)}_{\sqrt{N} \phi _+(h)} \left( b(g) \right) ] \\&= \; (2^{2n-1} - 1) \Vert h \Vert _2^{2n-2} \langle g ,h \rangle \sqrt{N} [ \phi _+ (h) , i \phi _- (h) ] \\&\quad + \Vert h \Vert _2^{2n} \sqrt{N} [ \phi _+ (h) , b(g) ] \\&\quad - \Vert h \Vert ^{2n-2}_2 \langle g,h \rangle \sqrt{N} [ \phi _+ (h) , b^* (h) ] \; . \end{aligned}$$

With (2.1), (2.2), we find (using the identities $-2(2^{2n-1} - 1) - 2 = - 2^{2n}$ and $2 (2^{2n-1} - 1) + 1 = 2^{2n} - 1$),

$$\begin{aligned} \mathrm {ad}^{(2n+1)}_{\sqrt{N} \phi _+(h)} \left( b(g) \right)&= - 2^{2n} \sqrt{N} \Vert h \Vert _2^{2n} \langle g ,h \rangle \left( 1 - \frac{{\mathcal {N}}_+}{N} \right) \\&\quad + (2^{2n} -1) \frac{1}{\sqrt{N}} \Vert h \Vert _2^{2n-2} \langle g ,h \rangle a^* (h) a(h) \\&\quad + \frac{1}{\sqrt{N}} \Vert h \Vert _2^{2n} a^* (h) a (g) \end{aligned}$$

confirming (2.8). $$\square $$

### Proposition 2.2

Let $g, h \in L^2_{\perp \varphi } ({\mathbb {R}}^3)$. With the shorthand notation $\gamma _s = \cosh s$ and $\sigma _s = \sinh s$, we have

2.10 $$\begin{aligned}&\mathrm {e}^{\sqrt{N} \phi _+ (h)} b(g) \mathrm {e}^{-\sqrt{N} \phi _+(h)} \nonumber \\ {}&\quad =\; \gamma _{\Vert h \Vert } b(g) + \gamma _{\Vert h \Vert } \frac{\gamma _{\Vert h \Vert } - 1}{\Vert h \Vert ^2} \langle g , h \rangle i \phi _- (h) \nonumber \\ {}&\qquad - \frac{\gamma _{\Vert h \Vert } - 1}{\Vert h \Vert ^2} \langle g , h \rangle b^* (h) \nonumber \\ {}&\qquad - \sqrt{N} \, \gamma _{\Vert h \Vert } \frac{\sigma _{\Vert h \Vert }}{\Vert h \Vert } \langle g , h \rangle \left( 1 - \frac{{\mathcal {N}}_+}{N} \right) \nonumber \\ {}&\qquad + \frac{1}{\sqrt{N}} \, \frac{\sigma _{\Vert h \Vert }}{\Vert h \Vert } \frac{\gamma _{\Vert h \Vert } - 1}{ \Vert h \Vert ^2} \langle g , h \rangle a^* (h) a (h)\nonumber \\ {}&\qquad + \frac{1}{\sqrt{N}} \, \frac{\sigma _{\Vert h \Vert }}{\Vert h \Vert } a^* (h) a (g) \; . \end{aligned}$$

### Remark

A formula similar to (2.10) for $\mathrm{e}^{\sqrt{N} \phi _+ (h)} b^* (g) \mathrm{e}^{-\sqrt{N} \phi _+(h)}$ can be obtained by Hermitian conjugation of (2.10) (and replacing *h* by $-h$).

### Proof

The expressions (2.10) follow from the commutator expansion

2.11 $$\begin{aligned} \mathrm{e}^X \, Y \, \mathrm{e}^{-X} = \sum _{j=0}^\infty \frac{1}{j!} \, \mathrm {ad}_X^{(j)} (Y) \end{aligned}$$

combined with the formulas in Lemma 2.1. Since the operators $X = \sqrt{N} \phi _+ (h)$ and $Y = b (g)$ are bounded on the truncated Fock space ${\mathcal {F}}_+^{\le N}$, it is easy to show the validity of the expansion (2.11) for (2.10). (The difference between $\mathrm{e}^X Y \mathrm{e}^{-X}$ and $\sum _{j=0}^n \mathrm {ad}_X^{(j)} (Y) / j!$ converges to zero in norm, as $n \rightarrow \infty$, for every fixed $N \in {\mathbb {N}}$.) $\square$

In particular, it follows from (2.10) that, for $x \in {\mathbb {R}}^3$,

2.12 $$\begin{aligned}&\mathrm{e}^{\sqrt{N} \phi _+ (h)} b_x \mathrm{e}^{-\sqrt{N} \phi _+(h)} \nonumber \\&\quad =\; \gamma _{\Vert h \Vert } b_x + \gamma _{\Vert h \Vert } \frac{\gamma _{\Vert h \Vert } - 1}{\Vert h \Vert ^2} h(x) i \phi _- (h) \nonumber \\&\qquad - \frac{\gamma _{\Vert h \Vert } - 1}{\Vert h \Vert ^2} h (x) b^* (h) \nonumber \\&\qquad - \sqrt{N} \, \gamma _{\Vert h \Vert } \frac{\sigma _{\Vert h \Vert }}{\Vert h \Vert } h (x) \left( 1 - \frac{{\mathcal {N}}_+}{N} \right) \nonumber \\&\qquad + \frac{1}{\sqrt{N}} \, \frac{\sigma _{\Vert h \Vert }}{\Vert h \Vert } \frac{\gamma _{\Vert h \Vert } - 1}{ \Vert h \Vert ^2} h (x) a^* (h) a (h)\nonumber \\&\qquad + \frac{1}{\sqrt{N}} \, \frac{\sigma _{\Vert h \Vert }}{\Vert h \Vert } a^* (h) a_x \;. \end{aligned}$$

We will also need a formula for $\mathrm{e}^{\sqrt{N} \phi _+ (h)} a^*_x a_y \mathrm{e}^{-\sqrt{N} \phi _+(h)}$. To derive such an expression, we compute

$$\begin{aligned}&\frac{\mathrm{d}}{\mathrm{d}s} \mathrm{e}^{s \sqrt{N} \phi _+ (h)} a_x^* a_y \mathrm{e}^{-s \sqrt{N} \phi _+ (h)} \\&\quad = \sqrt{N} \mathrm{e}^{s \sqrt{N} \phi _+ (h)} [ \phi _+ (h) , a_x^* a_y ] \mathrm{e}^{-s \sqrt{N} \phi _+ (h)} \\&\quad = \sqrt{N} \, \overline{h(x)} \mathrm{e}^{s \sqrt{N} \phi _+ (h)} b_y \mathrm{e}^{-s \sqrt{N} \phi _+ (h)}\\&\qquad - \sqrt{N} h (y) \mathrm{e}^{s \sqrt{N} \phi _+ (h)} b^*_x \mathrm{e}^{-s \sqrt{N} \phi _+ (h)} \; . \end{aligned}$$

Using (2.12) (and its Hermitian conjugate) and then integrating over $s \in [0;1]$, we arrive at

2.13 $$\begin{aligned} \begin{aligned} \mathrm{e}^{s \sqrt{N} \phi _+ (h)}&a_x^* a_y \mathrm{e}^{-s \sqrt{N} \phi _+ (h)} \\ = \;&a_x^* a_y + \sqrt{N} \, \frac{\sigma _{\Vert h \Vert }}{\Vert h \Vert } \left( \overline{h(x)} \, b_y - h(y) \, b_x^* \right) \\&- N \frac{\sigma _{\Vert h \Vert }^2}{\Vert h \Vert ^2} \, \overline{h(x)} h(y) \, \left( 1- \frac{{\mathcal {N}}_+}{N} \right) + \frac{(\gamma _{\Vert h \Vert }-1)}{\Vert h \Vert ^2} \left( \overline{h(x)} a^* (h) a_y + h (y) a_x^* a (h) \right) \\&+ \sqrt{N} \frac{\sigma _{\Vert h \Vert }}{\Vert h \Vert } \frac{(\gamma _{\Vert h \Vert }-1)}{\Vert h \Vert ^2} \, \overline{h(x)} h(y) \, i \phi _- (h) + \left( \frac{\gamma _{\Vert h \Vert }-1}{\Vert h \Vert ^2} \right) ^2 \overline{h(x)} h(y) \, a^* (h) a (h)\; . \end{aligned} \nonumber \\\end{aligned}$$

Integrating (2.13) against the integral kernel of a self-adjoint operator, we can also get a formula for $\mathrm{e}^{\sqrt{N} \phi _+ (h)} \mathrm{d}\Gamma (H) \mathrm{e}^{-\sqrt{N} \phi _+(h)}$, for a self-adjoint operator *H*.

### Proposition 2.3

Let $H : D(H) \rightarrow L^2_{\perp \varphi } ({\mathbb {R}}^3)$ be self-adjoint, with $D(H) \subset L^2_{\perp \varphi } ({\mathbb {R}}^3)$ denoting the domain of *H*. Let $h \in D (H)$. Then

2.14 $$\begin{aligned}&\mathrm{e}^{\sqrt{N} \phi _+(h)} \mathrm {d}\Gamma (H) \mathrm{e}^{-\sqrt{N} \phi _+(h)} \nonumber \\&\quad = \; \mathrm {d}\Gamma (H) + \sqrt{N} \, \frac{\sigma _{\Vert h \Vert }}{\Vert h \Vert } i \phi _- (Hh) \nonumber \\&\qquad - N \frac{\sigma _{\Vert h \Vert }^2}{\Vert h \Vert ^2} \langle h, H h \rangle \left( 1 - \frac{{\mathcal {N}}_+}{N} \right) \nonumber \\&\qquad + \frac{(\gamma _{\Vert h \Vert } -1)}{\Vert h \Vert ^2} (a^* (h) a (Hh) + a^* (Hh) a (h) ) \nonumber \\&\qquad + \sqrt{N} \, \frac{\sigma _{\Vert h \Vert }}{\Vert h \Vert } \frac{\gamma _{\Vert h \Vert } - 1}{\Vert h \Vert ^2} \langle h, H h \rangle i \phi _- (h) \nonumber \\&\qquad + \left( \frac{\gamma _{\Vert h \Vert } - 1}{\Vert h \Vert ^2} \right) ^2 \langle h, H h \rangle a^* (h) a (h) \; . \end{aligned}$$

### Proposition 2.4

Let $h \in L^2_{\perp \varphi } ({\mathbb {R}}^3)$ and denote by ${\mathcal {N}}_+$ the number of particles operator on ${\mathcal {F}}_{\perp \varphi }^{\le N}$. Then, for every $s \in {\mathbb {R}}$,

2.15 $$\begin{aligned} \begin{aligned} \mathrm{e}^{-s {\mathcal {N}}_+} b (h) \mathrm{e}^{s {\mathcal {N}}_+}&= \mathrm{e}^s b(h) , \\ \mathrm{e}^{-s {\mathcal {N}}_+} b^* (h) \mathrm{e}^{s {\mathcal {N}}_+}&= \mathrm{e}^{-s} b^* (h) , \\ \mathrm{e}^{-s {\mathcal {N}}_+} \phi _+ (h) \mathrm{e}^{s {\mathcal {N}}_+}&= \cosh (s) \phi _+ (h) + \sinh (s) i \phi _- (h) , \\ \mathrm{e}^{-s {\mathcal {N}}_+} i \phi _- (h) \mathrm{e}^{s {\mathcal {N}}_+}&= \cosh (s) i \phi _- (h) + \sinh (s) \phi _+ (h) \; . \end{aligned} \end{aligned}$$

### Proof

From $[b (h) , {\mathcal {N}}_+] = b(h)$ and $[b^* (h), {\mathcal {N}}_+] = - b^* (h)$, we easily find that

$$\begin{aligned} \begin{aligned} \mathrm{e}^{-s {\mathcal {N}}_+} b (h) \mathrm{e}^{s {\mathcal {N}}_+}&= \mathrm{e}^s b(h), \\ \mathrm{e}^{-s {\mathcal {N}}_+} b^* (h) \mathrm{e}^{s {\mathcal {N}}_+}&= \mathrm{e}^{-s} b^* (h) \; .\end{aligned} \end{aligned}$$

Thus

$$\begin{aligned} \begin{aligned} \mathrm{e}^{-s {\mathcal {N}}_+} \phi _+ (h) \mathrm{e}^{s {\mathcal {N}}_+}&= \mathrm{e}^s b(h) + \mathrm{e}^{-s} b^* (h) ,\\ \mathrm{e}^{-s {\mathcal {N}}_+} i\phi _- (h) \mathrm{e}^{s {\mathcal {N}}_+}&= \mathrm{e}^s b(h) - \mathrm{e}^{-s} b^* (h) \; . \end{aligned} \end{aligned}$$

Writing $b(h) = (\phi _+ (h) + i\phi _- (h) )/2$ and $b^* (h) = (\phi _+ (h) - i\phi _- (h))/2$, we arrive at (2.15). $\square$

### Proposition 2.5

Let $t \mapsto \varphi _t$ with $\Vert \varphi _t \Vert _2 = 1$, independently of *t*. Let $t \mapsto h_t$ be a differentiable map, with values in $L^2_{\perp \varphi _t} ({\mathbb {R}}^3)$. For $\xi _1, \xi _2 \in {\mathcal {F}}_{\perp \varphi _t}^{\le N}$ we find

2.16 $$\begin{aligned}&\Big \langle \xi _1 , \Big [ \partial _t \mathrm{e}^{\sqrt{N} \phi _+ (h_t)} \Big ] \mathrm{e}^{-\sqrt{N} \phi _+ (h_t)} \xi _2 \Big \rangle \nonumber \\&\quad = \; \sqrt{N} \, \frac{\sigma _{\Vert h_t \Vert }}{\Vert h_t \Vert } \langle \xi _1 , \phi _+ ( \partial _t h_t) \xi _2 \rangle \nonumber \\&\qquad - \sqrt{N} \frac{\sigma _{\Vert h_t \Vert }}{\Vert h_t \Vert } \frac{\gamma _{\Vert h_t \Vert } -1}{\Vert h_t \Vert ^2} \text {Im} \langle \partial _t h_t , h_t \rangle \langle \xi _1, \phi _- (h_t) \xi _2 \rangle \nonumber \\&\qquad - \sqrt{N} \, \frac{\sigma _{\Vert h_t \Vert } - \Vert h_t \Vert }{\Vert h_t \Vert ^3} \text {Re} \langle \partial _t h_t , h_t \rangle \langle \xi _1, \phi _+ (h_t) \xi _2 \rangle \nonumber \\&\qquad - i N \frac{\sigma _{\Vert h_t \Vert }^2}{\Vert h_t \Vert ^2} \text {Im} \langle \partial _t h_t , h_t \rangle \langle \xi _1 , (1 - {\mathcal {N}}_+ / N) \xi _2 \rangle \nonumber \\&\qquad +i \left( \frac{\gamma _{\Vert h_t \Vert } - 1}{\Vert h_t \Vert ^2} \right) ^2 \text {Im} \langle \partial _t h_t , h_t \rangle \langle \xi _1 , a^* (h_t ) a(h_t) \xi _2 \rangle \nonumber \\&\qquad + \frac{\gamma _{\Vert h_t \Vert } - 1}{\Vert h_t \Vert ^2} \Big \langle \xi _1, \left[ a^* (h_t) a (\partial _t h_t) - a^* (\partial _t h_t) a (h_t) \right] \xi _2 \Big \rangle \; . \end{aligned}$$

### Proof

For any two bounded operators *A*, *B* we can write

$$\begin{aligned} \mathrm{e}^A - \mathrm{e}^B = \left[ \mathrm{e}^A \mathrm{e}^{-B} - 1 \right] \mathrm{e}^B = \left[ \int _0^1 \mathrm{d}\tau \, \frac{\mathrm{d}}{\mathrm{d}\tau } \mathrm{e}^{\tau A} \mathrm{e}^{-\tau B} \right] \mathrm{e}^B = \int _0^1 \mathrm{d}\tau \, \mathrm{e}^{\tau A} (A-B) \mathrm{e}^{(1-\tau ) B} \; . \end{aligned}$$

Hence, if $t \rightarrow A_t$ is an operator-valued functions, differentiable in *t*, we find

$$\begin{aligned} \mathrm{e}^{A_{t+h}} - \mathrm{e}^{A_t} = \int _0^1 \mathrm{d}\tau \, \mathrm{e}^{\tau A_{t+h}} (A_{t+h} - A_t) \mathrm{e}^{(1-\tau )A_t} \end{aligned}$$

Dividing by *h* and letting $h \rightarrow 0$, we find

$$\begin{aligned} \partial _t \mathrm{e}^{A_t} = \int _0^1 \mathrm{d}\tau \, \mathrm{e}^{\tau A_t} \partial _t A_t \mathrm{e}^{(1-\tau ) A_t} \; . \end{aligned}$$

In particular,

$$\begin{aligned} \left[ \partial _t \mathrm{e}^{\sqrt{N} \phi _+ (h_t)} \right] \mathrm{e}^{-\sqrt{N} \phi _+ (h_t)} = \sqrt{N} \int _0^1 \mathrm{d}\tau \, \mathrm{e}^{\tau \sqrt{N} \phi _+ (h_t)} \phi _+ ( \partial _t h_t) \mathrm{e}^{-\tau \sqrt{N} \phi _+ (h_t)} \; . \end{aligned}$$

With Prop. 2.2, we find

$$\begin{aligned}&\Big [ \partial _t \mathrm{e}^{\sqrt{N} \phi _+ (h_t)} \Big ] \mathrm{e}^{-\sqrt{N} \phi _+ (h_t)} \\&\quad = \; \sqrt{N} \int _0^1 \mathrm{d}\tau \left[ \gamma _{\tau \Vert h_t \Vert } \phi _+ ( \partial _t h_t) -2 \gamma _{\tau \Vert h_t \Vert } \frac{\gamma _{\tau \Vert h_t \Vert } - 1}{\Vert h_t \Vert ^2} \text {Im} \langle \partial _t h_t , h_t \rangle \phi _- (h_t) \right. \\&\qquad - \frac{\gamma _{\tau \Vert h_t \Vert } -1}{\Vert h_t \Vert ^2} \text {Re} \langle \partial _t h_t , h_t \rangle \phi _+ (h_t) - \frac{\gamma _{\tau \Vert h_t \Vert } -1}{\Vert h_t \Vert ^2} \text {Im} \langle \partial _t h_t , h_t \rangle \phi _- (h_t) \\&\qquad -2i \sqrt{N} \gamma _{\tau \Vert h_t \Vert } \frac{\sigma _{\tau \Vert h_t \Vert }}{\Vert h_t \Vert } \text {Im} \langle \partial _t h_t , h_t \rangle (1 - {\mathcal {N}}_+ / N)\\&\qquad + \frac{2i}{\sqrt{N}} \frac{\sigma _{\tau \Vert h_t \Vert }}{\Vert h_t \Vert } \frac{\gamma _{\tau \Vert h_t \Vert } - 1}{\Vert h_t \Vert ^2} \text {Im } \langle \partial _t h_t , h_t \rangle a^* (h_t ) a(h_t) \\&\qquad \left. + \frac{1}{\sqrt{N}} \frac{\sigma _{\tau \Vert h_t \Vert }}{\Vert h_t \Vert } \left[ a^* (h_t) a (\partial _t h_t) - a^* (\partial _t h_t) a (h_t) \right] \right] \; . \end{aligned}$$

Integrating over $\tau$, we arrive at (2.16). $\square$

## Proof of main theorem

To prove Theorem 1.1, we start from (1.15), writing

$$\begin{aligned}&{\mathbb {E}}_{\psi _{N,t}} \, \mathrm{e}^{\lambda \left[ \sum _{j=1}^N (O^{(j)} - \langle \varphi _t , O \varphi _t \rangle )\right] } \\&\quad = \left\langle \Omega , {\mathcal {W}}^*_N (t;0) \mathrm{e}^{\lambda \mathrm{d}\Gamma (q_t \widetilde{O}_t q_t) + \lambda \sqrt{N} \phi _+ (q_t O \varphi _t )} {\mathcal {W}}_N (t;0) \Omega \right\rangle \; . \end{aligned}$$

### Lemma 3.1

There exist constants $C, c > 0$ such that

3.1 $$\begin{aligned}&\displaystyle \left\langle \Omega , {\mathcal {W}}^*_N (t;0) \mathrm {e}^{\lambda \mathrm {d}\Gamma (q_t \widetilde{O}_t q_t) + \lambda \sqrt{N} \phi _+ (q_t O \varphi _t )} {\mathcal {W}}_N (t;0) \Omega \right\rangle \nonumber \\ {}&\quad \le \mathrm {e}^{C N \Vert O \Vert ^3 \lambda ^3}\nonumber \\ {}&\qquad \times \left\langle \Omega , {\mathcal {W}}^*_N (t;0) \mathrm {e}^{\lambda \sqrt{N} \phi _+ (q_t O \varphi _t)/2} \mathrm {e}^{c \lambda \Vert O \Vert {\mathcal {N}}_+ (t)} \mathrm {e}^{\lambda \sqrt{N} \phi _+ (q_t O \varphi _t)/2} {\mathcal {W}}_N (t;0) \Omega \right\rangle \nonumber \\ \end{aligned}$$

for all $$\lambda \le \Vert O \Vert ^{-1}$$.

### Proof

For $s \in [0;1]$ and a fixed $\kappa > 0$, we define

$$\begin{aligned} \xi _{s} = \mathrm{e}^{(1-s) \lambda \kappa {\mathcal {N}}_+ (t) / 2} \mathrm{e}^{(1-s) \lambda \sqrt{N} \phi _+ (q_t O \varphi _t)/2} \mathrm{e}^{s \lambda [ \mathrm{d}\Gamma (q_t \widetilde{O}_t q_t ) + \sqrt{N} \phi _+ (q_t O \varphi _t) ]/2} {\mathcal {W}}_{N} (t;0) \Omega \; . \end{aligned}$$

Note that $\xi _s \in {\mathcal {F}}_{\perp \varphi _t}^{\le N}$ for all $s \in [0;1]$. Then, we have

$$\begin{aligned} \Vert \xi _{0} \Vert ^2 = \left\langle \Omega , {\mathcal {W}}_N^* (t;0) \mathrm{e}^{\lambda \sqrt{N} \phi _+ (q_t O \varphi _t)/2} \mathrm{e}^{\lambda \kappa {\mathcal {N}}_+ (t)} \mathrm{e}^{\lambda \sqrt{N} \phi _+ (q_t O \varphi _t)/2} {\mathcal {W}}_N (t;0) \Omega \right\rangle \end{aligned}$$

and

$$\begin{aligned} \Vert \xi _{1} \Vert ^2 = \left\langle \Omega , {\mathcal {W}}_N^* (t;0) \mathrm{e}^{\lambda [ \mathrm{d}\Gamma (q_t \widetilde{O}_t q_t ) + \sqrt{N} \phi _+ (q_t O \varphi _t) ]} {\mathcal {W}}_N (t;0) \Omega \right\rangle \; . \end{aligned}$$

To compare $\Vert \xi _{1} \Vert ^2$ with $\Vert \xi _{0} \Vert ^2$, we compute the derivative

$$\begin{aligned} \partial _s \Vert \xi _{s} \Vert ^2 = 2\text {Re } \langle \xi _{s} ; \partial _s \xi _{s} \rangle \; . \end{aligned}$$

We have $\partial _s \xi _{s} = {\mathcal {M}}_{s} \xi _{s}$, with

$$\begin{aligned} {\mathcal {M}}_{s}= & {} \frac{\lambda }{2} \mathrm{e}^{(1-s) \lambda \kappa {\mathcal {N}}_+ (t) /2} \mathrm{e}^{(1-s) \lambda \sqrt{N} \phi _+ (q_t O \varphi _t) /2} \mathrm{d}\Gamma \\&(q_t \widetilde{O}_t q_t ) \mathrm{e}^{-(1-s) \lambda \sqrt{N} \phi _+ (q_t O \varphi _t) /2} \mathrm{e}^{-(1-s) \lambda \kappa {\mathcal {N}}_+ /2} - \frac{\lambda \kappa }{2} {\mathcal {N}}_+ (t) \; . \end{aligned}$$

With Proposition 2.3 we find, defining $h_t = (1-s) \lambda q_t O \varphi _t$,

$$\begin{aligned}&\mathrm{e}^{(1-s) \lambda \sqrt{N} \phi _+ (q_t O \varphi _t)} \mathrm {d}\Gamma (q_t \widetilde{O}_t q_t ) \mathrm{e}^{-(1-s) \lambda \sqrt{N} \phi _+ (q_t O \varphi _t)} \nonumber \\&\quad = \; \mathrm {d}\Gamma (q_t \widetilde{O}_t q_t) - N \frac{\sigma _{\Vert h_t \Vert }^2}{\Vert h_t \Vert ^2} \langle h_t , \widetilde{O}_t h_t \rangle \left( 1 - \frac{{\mathcal {N}}_+ (t)}{N} \right) \nonumber \\&\qquad + \left( \frac{\gamma _{\Vert h_t \Vert } - 1}{\Vert h_t \Vert ^2} \right) ^2 \langle h_t , \widetilde{O}_t h_t \rangle a^* (h_t) a (h_t) \nonumber \\&\qquad + \frac{\gamma _{\Vert h_t \Vert } -1}{\Vert h_t \Vert ^2} (a^* (h_t) a (q_t \widetilde{O}_t h_t) + a^* (q_t \widetilde{O}_t h_t) a (h_t) ) \nonumber \\&\qquad + \sqrt{N} \, \frac{\sigma _{\Vert h_t \Vert }}{\Vert h_t \Vert } \frac{\gamma _{\Vert h_t \Vert } - 1}{\Vert h_t \Vert ^2} \langle h_t , \widetilde{O}_t h_t \rangle i \phi _- (h_t) + \sqrt{N} \, \frac{\sigma _{\Vert h_t \Vert }}{\Vert h_t \Vert } i \phi _- (q_t \widetilde{O}_t h_t) \; . \end{aligned}$$

With Proposition 2.4, we obtain

$$\begin{aligned} \frac{2}{\lambda } \, {\mathcal {M}}_{s}= & {} \mathrm {d}\Gamma (q_t \widetilde{O}_t q_t) - N \frac{\sigma _{\Vert h_t \Vert }^2}{\Vert h_t \Vert ^2} \langle h_t , \widetilde{O}_t h_t \rangle \left( 1 - \frac{{\mathcal {N}}_+ (t)}{N} \right) \\&+ \left( \frac{\gamma _{\Vert h_t \Vert } - 1}{\Vert h_t \Vert ^2} \right) ^2 \langle h_t , \widetilde{O}_t h_t \rangle a^* (h_t) a (h_t) \\&+ \frac{\gamma _{\Vert h_t \Vert } -1}{\Vert h_t \Vert ^2} (a^* (h_t) a (q_t \widetilde{O}_t h_t) + a^* (q_t \widetilde{O}_t h_t) a (h_t) ) \\&+ \sqrt{N} \, \frac{\sigma _{\Vert h_t \Vert }}{\Vert h_t \Vert } \frac{\gamma _{\Vert h_t \Vert } - 1}{\Vert h_t \Vert ^2} \langle h_t , \widetilde{O}_t h_t \rangle \\&\left[ \cosh ((1-s)\lambda \kappa /2) i\phi _- (h_t) \right. \\&\left. +\, \sinh ((1-s) \lambda \kappa /2) \phi _+ (h_t) \right] \\&+ \sqrt{N} \, \frac{\sigma _{\Vert h_t \Vert }}{\Vert h_t \Vert } \left[ \cosh ((1-s) \lambda \kappa /2) i \phi _- (q_t \widetilde{O}_t h_t) + \sinh ((1-s) \lambda \kappa /2) \phi _+ (q_t \widetilde{O}_t h_t) \right] \\&\qquad - \kappa {\mathcal {N}}_+ (t) \; . \end{aligned}$$

Using the bounds (2.6), (2.7), and the fact that $\Vert h_t \Vert \le \lambda \Vert O \Vert \le 1$ (from the assumption $\lambda \le \Vert O \Vert ^{-1}$), we find

$$\begin{aligned} \begin{aligned} \frac{2}{\lambda } \text {Re } \langle \xi _{s} , \partial _s \xi _{s} \rangle = \;&\frac{2}{\lambda } \text {Re } \langle \xi _{s} , {\mathcal {M}}_{s} \xi _{s} \rangle \\ \le \;&\left[ C \Vert O \Vert - \kappa \right] \Vert {\mathcal {N}}_+^{1/2} (t) \xi _{s} \Vert ^2 + C \lambda ^2 N \Vert O \Vert ^3 \mathrm{e}^{\lambda \kappa } \Vert \xi _{s} \Vert ^2 \; .\end{aligned} \end{aligned}$$

Choosing $\kappa = c \Vert O \Vert$ (which also implies that $\lambda \kappa \le c$), we conclude that

$$\begin{aligned} \partial _s \Vert \xi _{N,s} \Vert ^2 \le C N \Vert O \Vert ^3 \lambda ^3 \Vert \xi _{N,s} \Vert ^2 \; . \end{aligned}$$

By Gronwall, we obtain (3.1). $\square$

### Lemma 3.2

For a bounded self-adjoint operator *O* on $L^2 ({\mathbb {R}}^3)$ with $\Vert \Delta O (1-\Delta )^{-1} \Vert _\text {op} < \infty$, we recall the notation ${\left| \left| \left| O \right| \right| \right| }$ from (1.13). Recall also that, for $0 \le s \le t$, $f_{s;t}$ denotes the solution of the equation (1.11). For given $c > 0$, there exists a constant $C > 0$ such that, with the definition

3.2 $$\begin{aligned} \kappa _s = c \Vert O \Vert _\text {op} \, \mathrm{e}^{C (\Vert v \Vert _1 + \Vert v \Vert _\infty ) s} + \frac{{\left| \left| \left| O \right| \right| \right| }}{\Vert v \Vert _1 + \Vert v \Vert _\infty } \left( \mathrm{e}^{C ( \Vert v \Vert _1 + \Vert v \Vert _\infty ) s} - 1 \right) . \end{aligned}$$

we have

$$\begin{aligned}&\left\langle \Omega , {\mathcal {W}}_N (t;0) \mathrm{e}^{\lambda \sqrt{N} \phi _+ (q_t O \varphi _t)/2} \mathrm{e}^{c \Vert O \Vert {\mathcal {N}}_+ (t)} \mathrm{e}^{\lambda \sqrt{N} \phi _+ (q_t O \varphi _t)/2} {\mathcal {W}}_N (t;0) \Omega \right\rangle \\&\quad \le \mathrm{e}^{C N \lambda ^3 {\left| \left| \left| O \right| \right| \right| }^3 \exp (C (1+ \Vert v \Vert _1 + \Vert v \Vert _\infty ) t)} \left\langle \Omega , \mathrm{e}^{\lambda \sqrt{N} \phi _+ (f_{0;t})/2} \mathrm{e}^{\lambda \kappa _t {\mathcal {N}}_+ (0)} \mathrm{e}^{\lambda \sqrt{N} \phi _+ (f_{0;t})/2} \Omega \right\rangle \end{aligned}$$

for all $$\lambda \le {\left| \left| \left| O \right| \right| \right| }^{-1} \mathrm{e}^{-C (\Vert v \Vert _\infty + \Vert v \Vert _{1} ) t}$$.

### Proof

For $s \in [0;t]$ and with $\kappa _s$ as in (3.2), we define

$$\begin{aligned} \xi _t (s) = \mathrm{e}^{\lambda \kappa _s {\mathcal {N}}_+ (s) /2} \mathrm{e}^{\lambda \sqrt{N} \phi _+ (f_{s;t}) /2} {\mathcal {W}}_N (s;0) \Omega \in {\mathcal {F}}_{\perp \varphi _s}^{\le N} \end{aligned}$$

With $\kappa _0 = c \Vert O \Vert$, we observe that

$$\begin{aligned} \Vert \xi _t (0) \Vert ^2 = \langle \Omega , \mathrm{e}^{\lambda \sqrt{N} \phi _+ (f_{0;t}) /2} \mathrm{e}^{c \lambda \Vert O \Vert {\mathcal {N}}_+ (0)} \mathrm{e}^{\lambda \sqrt{N} \phi _+ (f_{0;t}) /2} \Omega \rangle \; .\end{aligned}$$

and that

$$\begin{aligned} \Vert \xi _t (t) \Vert ^2 = \left\langle \Omega , {\mathcal {W}}_N (t;0)^* \mathrm{e}^{\lambda \sqrt{N} \phi _+ (q_t O \varphi _t) /2} \mathrm{e}^{\lambda \kappa _t {\mathcal {N}}_+ (t)} \mathrm{e}^{\lambda \sqrt{N} \phi _+ (q_t O \varphi _t) /2} {\mathcal {W}}_N (t;0) \Omega \right\rangle \, . \end{aligned}$$

To compare $\Vert \xi _t (0) \Vert ^2$ with $\Vert \xi _t (t) \Vert ^2$, we are going to compute the derivative with respect to *s*. Since the two norms are taken on different spaces, it is convenient to embed first the *s*-dependent space ${\mathcal {F}}_{\perp \varphi _s}^{\le N}$ into the full, *s*-independent, Fock space ${\mathcal {F}}= \bigoplus _{n \ge 0} L^2 ({\mathbb {R}}^{3n})^{\otimes _s n}$. To this end, we observe that

$$\begin{aligned} \begin{aligned} \Vert \xi _t (s) \Vert ^2 = \;&\left\langle \Omega , {\mathcal {W}}_N (s;0)^* \mathrm{e}^{\lambda \sqrt{N} \phi _+ (f_{s;t}) /2} \mathrm{e}^{\lambda \kappa _s {\mathcal {N}}_+ (s)} \mathrm{e}^{\lambda \sqrt{N} \phi _+ (f_{s;t}) /2} {\mathcal {W}}_N (s;0) \Omega \right\rangle _{{\mathcal {F}}} \\ = \;&\left\langle \Omega , {\mathcal {W}}_N (s;0)^* \mathrm{e}^{\lambda \sqrt{N} \phi _+ (f_{s;t}) /2} \mathrm{e}^{\lambda \kappa _s {\mathcal {N}}} \mathrm{e}^{\lambda \sqrt{N} \phi _+ (f_{s;t}) /2} {\mathcal {W}}_N (s;0) \Omega \right\rangle _{{\mathcal {F}}} \end{aligned} \end{aligned}$$

where ${\mathcal {N}}$ denotes now the number of particles operator on ${\mathcal {F}}$. Hence, we obtain

3.3 $$\begin{aligned} \partial _s \Vert \xi _t (s) \Vert ^2 = -i \left\langle \xi _t (s) ; \left[ {\mathcal {J}}_{N,t} (s) - {\mathcal {J}}^*_{N,t} (s) \right] \xi _t (s) \right\rangle \end{aligned}$$

with the generator (this formula holds if we interpret ${\mathcal {J}}_{N,t} (s)$ as a quadratic form on ${\mathcal {F}}_{\perp \varphi _s}^{\le N}$)

3.4 $$\begin{aligned} {\mathcal {J}}_{N,t} (s)= & {} \frac{i\lambda }{2} {\dot{\kappa }}_s \, {\mathcal {N}}_+ (s) + \mathrm{e}^{\lambda \kappa _s \, {\mathcal {N}}_+ (s) / 2}\nonumber \\&\quad \left[ i \partial _s \mathrm{e}^{\lambda \sqrt{N} \phi _+ (f_{s;t}) /2} \right] \mathrm{e}^{-\lambda \sqrt{N} \phi _+ (f_{s;t}) /2} \mathrm{e}^{-\lambda \kappa _s \, {\mathcal {N}}_+ (s) /2} \nonumber \\&\quad + \mathrm{e}^{\lambda \kappa _s {\mathcal {N}}_+ (s)/ 2} \mathrm{e}^{\lambda \sqrt{N} \phi _+ (f_{s;t}) /2} {\mathcal {L}}_N (s) \mathrm{e}^{-\lambda \sqrt{N} \phi _+ (f_{s;t}) /2} \mathrm{e}^{-\lambda \kappa _s {\mathcal {N}}_+ (s) / 2} \; .\qquad \quad \end{aligned}$$

Remark that only the antisymmetric part of ${\mathcal {J}}_{N,t} (s)$ contributes to the growth of the norm.

Next, we compute ${\mathcal {J}}_{N,t} (s)$, focusing in particular on its antisymmetric component. We recall the definition (1.6) of the generator ${\mathcal {L}}_N (s)$. We introduce the notation $h_{s;t} = \lambda f_{s;t} /2 \in L^2_{\perp \varphi _s} ({\mathbb {R}}^3)$. From (2.14), we find, on vectors in ${\mathcal {F}}_{\perp \varphi _s}^{\le N}$ (since we consider matrix elements on vectors in ${\mathcal {F}}_{\perp \varphi _s}^{\le N}$, we can replace the operator $h_H (s) + K_{1,s}$, which does not leave $L^2_{\perp \varphi _s} ({\mathbb {R}}^3)$ invariant, with its restriction to $L^2_{\perp \varphi _s} ({\mathbb {R}}^3)$; this is the reason why we can apply Prop. 2.3)

$$\begin{aligned}&\mathrm{e}^{\lambda \sqrt{N} \phi _+ (f_{s;t}) /2} \mathrm{d}\Gamma (h_H (s) + K_{1,s}) \mathrm{e}^{-\lambda \sqrt{N} \phi _+ (f_{s;t}) /2} \\&\quad = \mathrm{d}\Gamma (h_H (s) + K_{1,s}) + \sqrt{N} \, \frac{\sigma _{\Vert h_{s;t} \Vert }}{\Vert h_{s;t} \Vert } i \phi _- ((h_H (s) + K_{1,s}) h_{s;t}) \\&\qquad - N \frac{\sigma ^2_{\Vert h_{s;t} \Vert }}{\Vert h_{s;t} \Vert ^2} \langle h_{s;t} , (h_H (s) + K_{1,s}) h_{s;t} \rangle (1 - {\mathcal {N}}_+ (s) / N) \\&\qquad + \frac{\gamma _{\Vert h_{s;t} \Vert } - 1}{ \Vert h_{s;t} \Vert ^2} \left( a^* (h_{s;t}) a ((h_H (s) + K_{1,s}) h_{s;t}) + a^*((h_H (s) + K_{1,s}) h_{s;t}) a (h_{s;t}) \right) \\&\qquad + \sqrt{N} \frac{\sigma _{\Vert h_{s;t} \Vert }}{\Vert h_{s;t} \Vert } \frac{\gamma _{\Vert h_{s;t} \Vert } - 1}{\Vert h_{s;t} \Vert ^2} \langle h_{s;t} , (h_H (s) + K_{1,s}) h_{s;t} \rangle i \phi _- (h_{s;t}) \\&\qquad + \left( \frac{\gamma _{\Vert h_{s;t} \Vert } -1}{\Vert h_{s;t} \Vert ^2} \right) ^2 \langle h_{s;t} , (h_H (s) + K_{1,s}) h_{s;t} \rangle a^* (h_{s;t}) a (h_{s;t}) \; . \end{aligned}$$

With Prop. 2.4, we obtain, again in the sense of forms on ${\mathcal {F}}_{\perp \varphi _s}^{\le N}$,

$$\begin{aligned}&\mathrm {e}^{\lambda \kappa _s {\mathcal {N}}_+ (s)/ 2} \mathrm {e}^{\lambda \sqrt{N} \phi _+ (f_{s;t}) /2} \mathrm {d}\Gamma (h_H (s) + K_{1,s}) \mathrm {e}^{-\lambda \sqrt{N} \phi _+ (f_{s;t}) /2} \mathrm {e}^{-\lambda \kappa _s {\mathcal {N}}_+ (s) / 2} \\ {}&\quad = \; \mathrm {d}\Gamma (h_H (s) + K_{1,s}) \\ {}&\qquad + \sqrt{N} \, \frac{\sigma _{\Vert h_{s;t} \Vert }}{\Vert h_{s;t} \Vert } \left[ \cosh (\lambda \kappa _s /2) i \phi _- ((h_H (s) + K_{1,s}) h_{s;t})\right. \\ {}&\qquad \qquad \qquad \left. - \sinh (\lambda \kappa _s /2) \phi _+ ((h_H (s) + K_{1,s}) h_{s;t}) \right] \\ {}&\qquad - N \frac{\sigma ^2_{\Vert h_{s;t} \Vert }}{\Vert h_{s;t} \Vert ^2} \langle h_{s;t} , (h_H (s) + K_{1,s}) h_{s;t} \rangle (1 - {\mathcal {N}}_+ / N) \\ {}&\qquad + \frac{\gamma _{\Vert h_{s;t} \Vert } - 1}{ \Vert h_{s;t} \Vert ^2} \left( a^* (h_{s;t}) a ((h_H (s) + K_{1,s}) h_{s;t}) + a^*((h_H (s) + K_{1,s}) h_{s;t}) a (h_{s;t}) \right) \\ {}&\qquad + \sqrt{N} \frac{\sigma _{\Vert h_{s;t} \Vert }}{\Vert h_{s;t} \Vert } \frac{\gamma _{\Vert h_{s;t} \Vert } - 1}{\Vert h_{s;t} \Vert ^2} \langle h_{s;t} , (h_H (s) + K_{1,s}) h_{s;t} \rangle \\ {}&\qquad \qquad \times \left[ \cosh (\lambda \kappa _s /2) i \phi _- (h_{s;t}) - \sinh (\lambda \kappa _s/2) \phi _+ (h_{s;t}) \right] \\ {}&\qquad + \left( \frac{\gamma _{\Vert h_{s;t} \Vert } -1}{\Vert h_{s;t} \Vert ^2} \right) ^2 \langle h_{s;t} , (h_H (s) + K_{1,s}) h_{s;t} \rangle a^* (h_{s;t}) a (h_{s;t}) \; . \end{aligned}$$

Removing symmetric terms (which do not contribute to (3.3)) and focusing on terms that are at most quadratic in $\lambda$ (recall that $h_{s;t} = \lambda f_{s;t} /2$), we arrive at

3.5 $$\begin{aligned}&\mathrm{e}^{\lambda \kappa _s {\mathcal {N}}_+ (s) / 2} \mathrm{e}^{\lambda \sqrt{N} \phi _+ (f_{s;t}) /2} \mathrm{d}\Gamma (h_H (s) + K_{1,s}) \mathrm{e}^{-\lambda \sqrt{N} \phi _+ (f_{s;t}) /2} \mathrm{e}^{-\lambda \kappa _s {\mathcal {N}}_+ (s) / 2} \nonumber \\&\quad = \; \frac{i \lambda \sqrt{N}}{2} \phi _- ((h_H (s) + K_{1,s}) f_{s;t}) + S_1 + T_1 \end{aligned}$$

where $S_1 = S_1^*$ does not contribute to the antisymmetric part of ${\mathcal {J}}_{N,t} (s)$ and

$$\begin{aligned} \Vert T_1 \Vert _\text {op} \le C N ( {\left| \left| \left| O \right| \right| \right| } \mathrm{e}^{Ct} + \kappa _s)^3 \lambda ^3.\end{aligned}$$

for all $\lambda > 0$ with $\lambda \Vert O \Vert \le 1$ and $\lambda \kappa _s \le 1$ for all $s \in [0;t]$. Here, we used that

$$\begin{aligned} \Vert (h_H (s) + K_{1,s} ) f_{s;t} \Vert \le C {\left| \left| \left| O \right| \right| \right| } \mathrm{e}^{C t} \end{aligned}$$

for all $$s \in [0;t]$$, $$ t > 0$$. This follows from the estimate $$\Vert \varphi _t \Vert _{H^4} \le C \mathrm{e}^{C|t|}$$, for a constant $$C > 0$$ depending on $$\Vert \varphi \Vert _{H^4}$$ (propagation of high Sobolev norms for the Hartree equation is standard; see [9]).

To handle the quadratic off-diagonal term with kernel $K_{2,s}$ in (1.6), we apply (2.12) (and its Hermitian conjugate, with *h* replaced by $-h$, for $b_x^*$, $b_y^*$) and then Prop. 2.4. Removing the symmetric part and keeping track only of contributions that are at most quadratic in $\lambda$, we find

$$\begin{aligned}&\mathrm {e}^{\lambda \kappa _s {\mathcal {N}}_+ (s) / 2} \mathrm {e}^{\lambda \sqrt{N} \phi _+ (f_{s;t}) /2} \left( \int \left[ K_{2,s} (x;y) b_x b_y + {\overline{K}}_{2,s} (x;y) b_x^* b_y^* \right] \, \mathrm {d}x \mathrm {d}y \right) \\ {}&\quad \times \mathrm {e}^{-\lambda \sqrt{N} \phi _+ (f_{s;t}) /2} \mathrm {e}^{-\lambda \kappa _s {\mathcal {N}}_+ (s) / 2} \\ {}&\quad =\; - \lambda \kappa _s \int \left[ K_{2,s} (x;y) b_x b_y - {\overline{K}}_{2,s} (x;y) b^*_x b^*_y \right] \, \mathrm {d}x \mathrm {d}y \\ {}&\qquad - \lambda \sqrt{N} \left[ \left( 1- \frac{{\mathcal {N}}_+ (s) +1/2}{N} \right) b (\overline{K_{2;s} f_{s;t}})\right. \\ {}&\qquad \left. -b^* (\overline{K_{2;s} f_{s;t}}) \left( 1- \frac{{\mathcal {N}}_+ (s) +1/2}{N} \right) \right] \\ {}&\qquad + \frac{\lambda }{2\sqrt{N}} \left[ \int \mathrm {d}x \mathrm {d}y K_{2,s} (x;y) b^* (f_{s;t}) a_x a_y - \int \mathrm {d}x \mathrm {d}y {\overline{K}}_{2,s} (x,y) a^*_y a_x^* \, b (f_{s;t}) \right] \\ {}&\qquad + \frac{\lambda }{2\sqrt{N}} \left[ \int \mathrm {d}x \mathrm {d}y \, K_{2,s} (x;y) a^* (f_{s;t}) a_x b_y - \int \mathrm {d}x \mathrm {d}y \, {\overline{K}}_{2,s} (x;y) b_y^* a_x^* \, a(f_{s;t}) \right] \\ {}&\qquad + S_2 + T_2 \end{aligned}$$

where $S_2 = S_2^*$ and $\Vert T_2 \Vert _\text {op} \le C N ({\left| \left| \left| O \right| \right| \right| } + \kappa _s)^3 \lambda ^3$ for all $s \in [0;t]$, if $\lambda \Vert O \Vert \le 1$ and $\lambda \kappa _s \le 1$ for all $s \in [0;t]$. Thus, we obtain

3.6 $$\begin{aligned}&\mathrm {e}^{\lambda \kappa _s {\mathcal {N}}_+ (s) / 2} \mathrm {e}^{\lambda \sqrt{N} \phi _+ (f_{s;t}) /2}\left( \frac{1}{2} \int \left[ K_{2,s} (x;y) b_x b_y + {\overline{K}}_{2,s} (x;y) b_x^* b_y^* \right] \, \mathrm {d}x \mathrm {d}y \right) \nonumber \\ {}&\quad \times \mathrm {e}^{-\lambda \sqrt{N} \phi _+ (f_{s;t}) /2} \mathrm {e}^{-\lambda \kappa _s {\mathcal {N}}_+ (s) / 2} \nonumber \\ {}&\quad = - \frac{i \lambda \sqrt{N}}{2} \phi _- (\overline{K_{2;s} f_{s;t}}) + S_2 + T_2 + i R_2 \end{aligned}$$

where $$S_2 = S_2^*$$, $$\Vert T_2 \Vert _\text {op} \le C N ({\left| \left| \left| O \right| \right| \right| }+ \kappa _s)^3 \lambda ^3$$ and

$$\begin{aligned} \pm R_2 \le C (\kappa _s \Vert v \Vert _\infty + {\left| \left| \left| O \right| \right| \right| }) \lambda {\mathcal {N}}_+ (s) \end{aligned}$$

for all $s \in [0;t]$ and all $\lambda > 0$ with $\lambda \Vert O \Vert \le 1$ and $\lambda \kappa _s \le 1$ for all $s \in [0;t]$. (Here we used that $\Vert K_{2,s} \Vert _\text {op} \le \Vert K_{2,s} \Vert _\text {HS} \le \Vert v \Vert _\infty$ for all $s \in [0;t]$.)

Setting $d_s = (v * |\varphi _s|^2) + K_{1,s}$ and using Prop. 2.3 and then Prop. 2.4, we obtain

$$\begin{aligned}&\mathrm{e}^{\lambda \kappa _s {\mathcal {N}}_+ (s) / 2} \mathrm{e}^{\lambda \sqrt{N} \phi _+ (f_{s;t}) /2} \mathrm{d}\Gamma (d_s) ({\mathcal {N}}_+ (s) /N) \mathrm{e}^{-\lambda \sqrt{N} \phi _+ (f_{s;t}) /2} \mathrm{e}^{-\lambda \kappa _s {\mathcal {N}}_+ (s) / 2} \\&\quad = \frac{1}{2\sqrt{N}} \left[ \mathrm{d}\Gamma (d_s) i \phi _- (h_{s;t}) + i\phi _- (h_{s;t}) \mathrm{d}\Gamma (d_s) \right] \\&\qquad + \frac{1}{2\sqrt{N}} \left[ i\phi _- (d_s h_{s;t}) {\mathcal {N}}_+ + {\mathcal {N}}_+ i \phi _- (d_s h_{s;t}) \right] + S_3 + T_3 \end{aligned}$$

with $S_3^* = S_3$ and $\Vert T_3 \Vert _\text {op} \le C N ({\left| \left| \left| O \right| \right| \right| } + \kappa _s)^3 \lambda ^3$. We conclude that

3.7 $$\begin{aligned}&\mathrm{e}^{\lambda \kappa _s {\mathcal {N}}_+ (s) / 2} \mathrm{e}^{\lambda \sqrt{N} \phi _+ (f_{s;t}) /2} \mathrm{d}\Gamma (d_s) ({\mathcal {N}}_+ (s) /N) \mathrm{e}^{-\lambda \sqrt{N} \phi _+ (f_{s;t}) /2} \mathrm{e}^{-\lambda \kappa _s {\mathcal {N}}_+ (s) / 2}\nonumber \\&\quad = S_3 + T_3 + i R_3 \end{aligned}$$

where $$S_3 = S_3^*$$, $$\Vert T_3 \Vert _\text {op} \le C N ({\left| \left| \left| O \right| \right| \right| } + \kappa _s)^3 \lambda ^3$$ and

$$\begin{aligned} \pm R_3 \le C {\left| \left| \left| O \right| \right| \right| } \lambda {\mathcal {N}}_+ (s) \end{aligned}$$

for all $s \in [0;t]$ and all $\lambda > 0$ with $\lambda \Vert O \Vert \le 1$ and $\lambda \kappa _s \le 1$ for all $s \in [0;t]$.

We consider now

$$\begin{aligned} {\mathcal {C}} = \frac{1}{\sqrt{N}} \int \mathrm{d}x \mathrm{d}y \, v(x-y) \, \left[ b_x^* a_y^* a_x + a_x^* a_y b_x \right] \end{aligned}$$

Conjugating separately $b_x^*$ and $a_y^* a_x$ (or $a_x^* a_y$ and $b_x$ in the second term), we arrive, using (2.12) (and its Hermitian conjugate), (2.13) and then Prop. 2.4, at

$$\begin{aligned}&\mathrm{e}^{\lambda \kappa _s {\mathcal {N}}_+ (s) / 2} \mathrm{e}^{\sqrt{N} \phi _+ (h_{s;t})} {\mathcal {C}} \mathrm{e}^{-\sqrt{N} \phi _+ (h_{s;t})} \mathrm{e}^{- \lambda \kappa _s {\mathcal {N}}_+ (s) / 2} \\&\quad =\; \frac{\lambda \kappa _s}{2\sqrt{N}} \int \mathrm{d}x \mathrm{d}y \, v(x-y) \, \left[ b_x^* a_y^* a_x - a_x^* a_y b_x \right] \\&\qquad - \frac{\lambda }{2} \int \mathrm{d}x \mathrm{d}y \, v(x-y) \left[ f_{s;t} (y) b_x^* b_x - \overline{f_{s;t} (y)} b_x^* b_x \right] \\&\qquad - \frac{\lambda }{2} \int \mathrm{d}x \mathrm{d}y \, v(x-y) \left[ f_{s;t} (x) b_x^* b_y^* - \overline{f_{s;t} (x)} b_y b_x \right] \\&\qquad + \frac{\lambda }{2} \int \mathrm{d}x \mathrm{d}y \, v(x-y) \left[ \overline{f_{s;t} (x)} (1-{\mathcal {N}}_+ / N) a_y^* a_x - f_{s;t} (x) a_x ^* a_y (1-{\mathcal {N}}_+ /N) \right] \\&\qquad - \frac{\lambda }{2} \frac{1}{N} \int \mathrm{d}x \mathrm{d}y \, v(x-y) \left[ a_x^* a(f_{s;t} ) a_y^* a_x - a_x^* a_y a^* (f_{s;t}) a_x \right] + S_4 + T_4\end{aligned}$$

where $S_4^* = S_4$ and $\Vert T_4 \Vert _\text {op} \le C N ( {\left| \left| \left| O \right| \right| \right| } + \kappa _s)^3 \lambda ^3$, for all $s \in [0;t]$ and all $\lambda > 0$ with $\lambda \Vert O \Vert \le 1$ and $\lambda \kappa _s \le 1$ for all $s \in [0;t]$. We obtain that

3.8 $$\begin{aligned} \mathrm{e}^{\lambda \kappa _s {\mathcal {N}}_+ (s) / 2} \mathrm{e}^{\sqrt{N} \phi _+ (h_{s;t})} {\mathcal {C}} \mathrm{e}^{-\sqrt{N} \phi _+ (h_{s;t})} \mathrm{e}^{- \lambda \kappa _s {\mathcal {N}}_+ (s) / 2} = S_4 + T_4 + i R_4 \end{aligned}$$

where $$S_4 = S_4^*$$, $$\Vert T_4 \Vert _\text {op} \le C N ( {\left| \left| \left| O \right| \right| \right| } + \kappa _s)^3 \lambda ^3$$ and

$$\begin{aligned} \pm R_4 \le C ({\left| \left| \left| O \right| \right| \right| } + (\Vert v \Vert _1 + \Vert v \Vert _\infty ) \kappa _s) \lambda {\mathcal {N}}_+ (s) \, .\end{aligned}$$

Finally, we consider the term

$$\begin{aligned} {\mathcal {V}} = \frac{1}{2N} \int \mathrm{d}x \mathrm{d}y \, v(x-y) a_x^* a_y^* a_y a_x = \frac{1}{2N} \int \mathrm{d}x \mathrm{d}y \, v (x-y) a_x^* a_x a_y^* a_y - \frac{v(0)}{2N} {\mathcal {N}}_+ (s) \; .\end{aligned}$$

Conjugating separately $a_x^* a_x$ and $a_y^* a_y$ (and also the operator ${\mathcal {N}}_+ (s)$, using Prop. 2.3), we obtain

$$\begin{aligned}&\mathrm{e}^{\lambda \kappa _s {\mathcal {N}}_+ (s) / 2} \mathrm{e}^{\lambda \sqrt{N} \phi _+ (f_{s;t})/2} {\mathcal {V}} \mathrm{e}^{- \lambda \sqrt{N} \phi _+ (f_{s;t})/2} \mathrm{e}^{- \lambda \kappa _s {\mathcal {N}}_+ (s) / 2} \\&\quad = \frac{\lambda }{2\sqrt{N}} \int \mathrm{d}x \mathrm{d}y \, v(x-y) \, \left[ a_x^* a_x \overline{f_{s;t} (y)} b_y - b_y^* f_{s;t} (y) a_x^* a_x \right] +S_5 + T_5 \end{aligned}$$

where $S_5 = S_5^*$ and $\Vert T_5 \Vert _\text {op} \le C N ( {\left| \left| \left| O \right| \right| \right| } + \kappa _s)^3 \lambda ^3$. Thus

3.9 $$\begin{aligned} \mathrm{e}^{\lambda \kappa _s {\mathcal {N}}_+ (s) / 2} \mathrm{e}^{\lambda \sqrt{N} \phi _+ (f_{s;t})/2} {\mathcal {V}} \mathrm{e}^{- \lambda \sqrt{N} \phi _+ (f_{s;t})/2} \mathrm{e}^{- \lambda \kappa _s {\mathcal {N}}_+ (s) / 2} = S_5 + T_5 + iR_5 \end{aligned}$$

with $$S_5 = S_5^*$$, $$\Vert T_5 \Vert _\text {op} \le C N ( {\left| \left| \left| O \right| \right| \right| } + \kappa _s)^3 \lambda ^3$$ and

$$\begin{aligned} \pm R_5 \le C {\left| \left| \left| O \right| \right| \right| } \lambda {\mathcal {N}}_+ (s) \end{aligned}$$

for all $s \in [0;t]$ and all $\lambda > 0$ with $\lambda \Vert O \Vert \le 1$ and $\lambda \kappa _s \le 1$ for all $s \in [0;t]$.

Combining (3.5), (3.6), (3.7), (3.8) and (3.9), we conclude that

$$\begin{aligned}&\mathrm{e}^{\lambda \kappa _s {\mathcal {N}}_+ (s) / 2} \mathrm{e}^{\sqrt{N} \phi _+ (h_{s;t})} {\mathcal {L}}_N (s) \mathrm{e}^{-\sqrt{N} \phi _+ (h_{s;t})} \mathrm{e}^{- \lambda \kappa _s {\mathcal {N}}_+ (s) / 2} \\&\quad = \frac{i \lambda \sqrt{N}}{2} \phi _- ((h_H (s) + K_{1,s} + J K_{2,s}) f_{s;t}) + S +T + i R \end{aligned}$$

where $$S^* = S$$, $$\Vert T \Vert _\text {op} \le C N ( {\left| \left| \left| O \right| \right| \right| } \mathrm{e}^{Ct} + \kappa _s)^3 \lambda ^3$$ and

$$\begin{aligned} \pm R \le C \lambda ({\left| \left| \left| O \right| \right| \right| } + (\Vert v \Vert _\infty + \Vert v \Vert _1) \kappa _s) {\mathcal {N}}_+ (s) \end{aligned}$$

for all $s \in [0;t]$ and all $\lambda > 0$ with $\lambda \Vert O \Vert \le 1$ and $\lambda \kappa _s \le 1$ for all $s \in [0;t]$.

Let us now focus on the second term on the r.h.s. of (3.4). With Prop. 2.5 we find, in the sense of forms on ${\mathcal {F}}_{\perp \varphi _s}^{\le N}$ and keeping track only of contributions that are antisymmetric and at most quadratic in $\lambda$,

$$\begin{aligned}&\mathrm{e}^{\lambda \kappa _s {\mathcal {N}}_+ (s) / 2} \left[ i \partial _s \mathrm{e}^{\lambda \sqrt{N} \phi _+ (f_{s;t}) /2} \right] \mathrm{e}^{-\lambda \sqrt{N} \phi _+ (f_{s;t}) /2} \mathrm{e}^{-\lambda \kappa _s {\mathcal {N}}_+ (s) /2} \\&\quad = - \frac{i \lambda \sqrt{N}}{2} \, \phi _- ( i \partial _s f_{s;t}) + \widetilde{S} + \widetilde{T} \end{aligned}$$

where $$\widetilde{S} = \widetilde{S}^*$$ and $$\Vert \widetilde{T} \Vert _\text {op} \le C N ( {\left| \left| \left| O \right| \right| \right| }+ \kappa _s)^3 \lambda ^3$$.

From (1.11) and (3.4), we conclude that

$$\begin{aligned}&\pm \frac{1}{i} \left[ {\mathcal {J}}_{N,t} (s) - {\mathcal {J}}_{N,t}^* (s) \right] \le \; C N ( {\left| \left| \left| O \right| \right| \right| } \mathrm{e}^{Ct} + \kappa _s)^3 \lambda ^3\\&\quad + \lambda \left[ C ({\left| \left| \left| O \right| \right| \right| } + ( \Vert v \Vert _\infty + \Vert v \Vert _1) \kappa _s) - {\dot{\kappa }}_s \right] {\mathcal {N}}_+ (s) \end{aligned}$$

for all $s \in [0;t]$ and all $\lambda > 0$ with $\lambda \Vert O \Vert \le 1$ and $\lambda \kappa _s \le 1$ for all $s \in [0;t]$.

With the choice (3.2), we find

$$\begin{aligned} \begin{aligned} \pm \frac{1}{i} \left[ {\mathcal {J}}_{N,t} (s) - {\mathcal {J}}_{N,t}^* (s) \right] \le&\; C N ( {\left| \left| \left| O \right| \right| \right| } \mathrm {e}^{Ct} + \kappa _s)^3 \lambda ^3\\ {}&\quad + \lambda \left[ C ({\left| \left| \left| O \right| \right| \right| } + ( \Vert v \Vert _\infty + \Vert v \Vert _1) \kappa _s) - {\dot{\kappa }}_s \right] {\mathcal {N}}_+ (s) \end{aligned} \end{aligned}$$

for all $s \in [0;t]$ and all $\lambda \le C {\left| \left| \left| O \right| \right| \right| }^{-1} \mathrm{e}^{-C (\Vert v \Vert _1 + \Vert v \Vert _\infty ) t}$.

Inserting in (3.3), we obtain that

$$\begin{aligned} \left| \partial _s \Vert \xi _t (t) \Vert ^2 \right| \le C N \lambda ^3 {\left| \left| \left| O \right| \right| \right| } \mathrm{e}^{C (1+ \Vert v \Vert _1 + \Vert v \Vert _\infty ) t} \, \Vert \xi _t (s) \Vert ^2 \; .\end{aligned}$$

By Gronwall, we arrive at

$$\begin{aligned} \Vert \xi _t (t) \Vert ^2 \le \mathrm{e}^{C N \lambda ^3 {\left| \left| \left| O \right| \right| \right| }^3 \exp (C (1+ \Vert v \Vert _1 + \Vert v \Vert _\infty ) t)} \, \Vert \xi _t (0) \Vert ^2 \end{aligned}$$

for all $s \in [0;t]$ and all $\lambda \le {\left| \left| \left| O \right| \right| \right| }^{-1} \mathrm{e}^{-C (\Vert v \Vert _1 + \Vert v \Vert _\infty ) t}$. $\square$

### Lemma 3.3

Let $\kappa _t$ be defined as in (3.2). Then, there exists a constant $C > 0$ such that

3.10 $$\begin{aligned}&\left\langle \Omega , \mathrm{e}^{\lambda \sqrt{N} \phi _+ (f_{0;t})/2} \mathrm{e}^{\lambda \kappa _t {\mathcal {N}}_+ (0)} \mathrm{e}^{\lambda \sqrt{N} \phi _+ (f_{0;t})/2} \Omega \right\rangle \nonumber \\&\quad \le \mathrm{e}^{\lambda ^2 N \Vert f_{0;t} \Vert ^2/2} \mathrm{e}^{C N \lambda ^3 {\left| \left| \left| O \right| \right| \right| }^3 \exp (C (\Vert v \Vert _1 + \Vert v \Vert _\infty ) t)} \end{aligned}$$

for all $\lambda \le {\left| \left| \left| O \right| \right| \right| }^{-1} \mathrm{e}^{-C ( \Vert v \Vert _\infty + \Vert v \Vert _1) t}$ and all $t > 0$.

### Remark

The lemma could be extended to bound the expectation on the l.h.s. of (3.10) for a larger class of states, including quasi-free states, rather than only in the vacuum. This would allow us to consider more general initial data in Theorem 1.1. To keep the focus on the main novelty of our paper (the possibility of proving a large deviation principle for many-body quantum dynamics), we restricted our attention on the simplest case of factorized initial data (leading to the vacuum in (3.10).

### Proof

For $s \in [0;1]$ and setting $h_t = \lambda f_{0;t}/2 \in L^2_{\perp \varphi } ({\mathbb {R}}^3)$, we define

$$\begin{aligned} \xi _s = \mathrm{e}^{\lambda \kappa _t {\mathcal {N}}_+ (0) /2} \mathrm{e}^{s \sqrt{N} \phi _+ (h_t)} \mathrm{e}^{(1-s) \sqrt{N} b^* (h_t)} \mathrm{e}^{(1-s) \sqrt{N} b(h_t)} \mathrm{e}^{(1-s)^2 N \Vert h_t \Vert ^2 /2} \Omega \; .\end{aligned}$$

Then

$$\begin{aligned} \Vert \xi _1 \Vert ^2 = \left\langle \Omega , \mathrm{e}^{\lambda \sqrt{N} \phi _+ (f_{0;t})/2} \mathrm{e}^{\lambda \kappa _t {\mathcal {N}}_+ (0)} \mathrm{e}^{\lambda \sqrt{N} \phi _+ (f_{0;t})/2} \Omega \right\rangle \end{aligned}$$

is the quantity we want to estimate, while

3.11 $$\begin{aligned} \Vert \xi _0 \Vert ^2 = \mathrm{e}^{N \Vert h_t \Vert ^2} \langle \mathrm{e}^{\sqrt{N} b^* (h_t)} \Omega , \mathrm{e}^{\lambda \kappa _t {\mathcal {N}}_+ (0)} \mathrm{e}^{\sqrt{N} b^* (h_t)} \Omega \rangle \end{aligned}$$

is going to give the bound on the r.h.s. of (3.10).

To compare $\Vert \xi _1 \Vert ^2$ with $\Vert \xi _0 \Vert ^2$, we compute the derivative

3.12 $$\begin{aligned} \partial _s \Vert \xi _s \Vert ^2 = 2 \text {Re } \langle \xi _s , {\mathcal {G}}_s \xi _s \rangle \end{aligned}$$

where

$$\begin{aligned} {\mathcal {G}}_s =&\; - (1-s) N \Vert h_t \Vert ^2 \\ {}&\quad + \sqrt{N} \mathrm {e}^{\lambda \kappa _t {\mathcal {N}}_+ (0) /2} \mathrm {e}^{s \sqrt{N} \phi _+ (h_t)} \\ {}&\qquad \times \left[ \phi _+ (h_t) - b^* (h_t) - \mathrm {e}^{(1-s) \sqrt{N} b^* (h_t)} b (h_t) \mathrm {e}^{-(1-s) \sqrt{N} b^* (h_t)} \right] \\ {}&\qquad \times \mathrm {e}^{-s\sqrt{N} \phi _+ (h_t)} \mathrm {e}^{-\lambda \kappa _t {\mathcal {N}}_+ (0) /2} \end{aligned}$$

is defined so that $\partial _s \xi _s = {\mathcal {G}}_s \xi _s$. With the commutation relations (2.1)-(2.4), we find the identity

$$\begin{aligned}&\mathrm {e}^{(1-s) \sqrt{N} b^* (h_t)} b(h_t) \mathrm {e}^{-(1-s) \sqrt{N} b^* (h_t)} \\\quad&= b(h_t) - \sqrt{N} \Vert h_t \Vert ^2 (1-s) \left( 1- \frac{{\mathcal {N}}_+ (0)}{N} \right) - \Vert h_t \Vert ^2 (1-s)^2 b^* (h_t) \\ {}&\quad + \frac{(1-s)}{\sqrt{N}} a^* (h_t) a(h_t) \; .\end{aligned}$$

Thus

$$\begin{aligned}&{\mathcal {G}}_s = - \mathrm {e}^{\lambda \kappa _t {\mathcal {N}}_+ (0) /2} \mathrm {e}^{s \sqrt{N} \phi _+ (h_t)} \\ {}&\quad \times \left[ (1-s) \Vert h_t \Vert ^2 {\mathcal {N}}_+ (0) + (1-s) a^* (h_t) a(h_t) - \sqrt{N} \Vert h_t \Vert ^2 (1-s)^2 b^* (h_t) \right] \\ {}&\quad \times \mathrm {e}^{-s \sqrt{N} \phi _+ (h_t)} \mathrm {e}^{-\lambda \kappa _t {\mathcal {N}}_+ (0) /2} \; . \end{aligned}$$

With Prop. 2.2 and Prop. 2.3, we obtain

$$\begin{aligned} {\mathcal {G}}_s = - (1-s) \Vert h_t \Vert ^2 {\mathcal {N}}_+ (0) - (1-s) a^* (h_t) a(h_t) + T \end{aligned}$$

where (using the definition (3.2) of $\kappa _t$)

$$\begin{aligned} \Vert T \Vert \le C N \lambda ^3 {\left| \left| \left| O \right| \right| \right| }^3 \mathrm{e}^{C (\Vert v \Vert _1 + \Vert v \Vert _\infty ) t}, \end{aligned}$$

for all $\lambda \le {\left| \left| \left| O \right| \right| \right| }^{-1} \mathrm{e}^{-C ( \Vert v \Vert _\infty + \Vert v \Vert _1) t}$. (This guarantees that $\lambda \kappa _t \le 1$ and $\lambda \Vert O \Vert \le 1$.) From (3.12), we obtain

$$\begin{aligned} \partial _s \Vert \xi _s \Vert ^2 \le C N \lambda ^3 {\left| \left| \left| O \right| \right| \right| }^3 \mathrm{e}^{C (\Vert v \Vert _1 + \Vert v \Vert _\infty ) t} \Vert \xi _s \Vert ^2 \end{aligned}$$

and thus that

$$\begin{aligned} \Vert \xi _1 \Vert ^2 \le \mathrm{e}^{CN \lambda ^3 {\left| \left| \left| O \right| \right| \right| }^3 \exp (C (\Vert v \Vert _1 + \Vert v \Vert _\infty ) t)} \Vert \xi _0 \Vert ^2 \end{aligned}$$

for all $$\lambda \le {\left| \left| \left| O \right| \right| \right| }^{-1} \mathrm{e}^{-C ( \Vert v \Vert _\infty + \Vert v \Vert _1) t}$$.

It remains to compute

$$\begin{aligned}&\Vert \xi _0 \Vert ^2 = \mathrm{e}^{N \Vert h_t \Vert ^2} \langle \mathrm{e}^{\sqrt{N} b^* (h_t)} \Omega , \mathrm{e}^{\lambda \kappa _t {\mathcal {N}}_+ (0) }\mathrm{e}^{\sqrt{N} b^* (h_t)} \Omega \rangle \\&\quad = \mathrm{e}^{N \Vert h_t \Vert ^2} \sum _{n=0}^N \frac{N^n}{(n!)^2} \mathrm{e}^{\lambda \kappa _t n} \Vert b^* (h_t)^n \Omega \Vert ^2 \; . \end{aligned}$$

Notice that

$$\begin{aligned} \Vert b^* (h_t)^n \Omega \Vert ^2=&{} \left\| a^* (h_t) (1- {\mathcal {N}}_+ (0) /N)^{1/2} a^* (h_t) (1-{\mathcal {N}}_+ (0)/N)^{1/2}\right. \\ {}&\qquad \left. \dots a^* (h_t) (1-{\mathcal {N}}_+ (0) /N)^{1/2} \Omega \right\| ^2 \\=&\left\| a^* (h_t)^n (1- ({\mathcal {N}}_+ (0) + n -1)/N)^{1/2} (1- ({\mathcal {N}}_+ (0) +n -2)/N)^{1/2} \right. \\ {}&\qquad \left. \dots (1- {\mathcal {N}}_+ (0) / N)^{1/2} \Omega \right\| ^2 \\ =&\frac{(N - (n-1)) \dots (N-1)}{N^{(n-1)}} \Vert a^* (h_t)^n \Omega \Vert ^2 \\ =&\frac{(N-1)!}{N^{(n-1)} (N-n)!} n! \Vert h_t \Vert ^{2n} \; . \end{aligned}$$

Therefore, recalling that $h_t = \lambda f_{0;t}/2$

$$\begin{aligned} \Vert \xi _0 \Vert ^2= & {} \mathrm{e}^{N \Vert h_t \Vert ^2} \sum _{n=0}^N {N \atopwithdelims ()n} \Vert h_t \Vert ^{2n} \mathrm{e}^{\lambda \kappa _t n} \\= & {} \mathrm{e}^{N \Vert h_t \Vert ^2} \, \left( 1 + \Vert h_t \Vert ^2 \mathrm{e}^{\lambda \kappa _t} \right) ^N \\\le & {} \mathrm{e}^{N \Vert h_t \Vert ^2 (1 + \mathrm{e}^{\lambda \kappa _t})} \le \mathrm{e}^{N \lambda ^2 \Vert f_{0;t} \Vert ^2 / 2} \mathrm{e}^{C N \lambda ^3 {\left| \left| \left| O \right| \right| \right| }^3 \exp (C (\Vert v \Vert _\infty + \Vert v \Vert _1) t)}\end{aligned}$$

for all $\lambda \le {\left| \left| \left| O \right| \right| \right| }^{-1} \mathrm{e}^{-C ( \Vert v \Vert _\infty + \Vert v \Vert _1) t}$. We conclude that

$$\begin{aligned}&\left\langle \Omega , \mathrm{e}^{\lambda \sqrt{N} \phi _+ (f_{0;t})/2} \mathrm{e}^{\lambda \kappa _t {\mathcal {N}}_+ (0)} \mathrm{e}^{\lambda \sqrt{N} \phi _+ (f_{0;t})/2} \Omega \right\rangle \\&\quad \le \mathrm{e}^{N \lambda ^2 \Vert f_{0;t} \Vert ^2 / 2} \mathrm{e}^{C N \lambda ^3 {\left| \left| \left| O \right| \right| \right| }^3 \exp (C (\Vert v \Vert _\infty + \Vert v \Vert _1) t)} \end{aligned}$$

for all $$\lambda \le {\left| \left| \left| O \right| \right| \right| }^{-1} \mathrm{e}^{-C ( \Vert v \Vert _\infty + \Vert v \Vert _1) t}$$. $$\square $$

### Proof of Theorem 1.1

Combining Lemma 3.1, Lemma 3.2 and Lemma 3.3, we arrive at

$$\begin{aligned}&\left\langle \Omega , {\mathcal {W}}^*_N (t;0) \mathrm{e}^{\lambda \mathrm{d}\Gamma (q_t \widetilde{O}_t q_t) + \lambda \sqrt{N} \phi _+ (q_t O \varphi _t )} {\mathcal {W}}_N (t;0) \Omega \right\rangle \\&\quad \le \mathrm{e}^{N \lambda ^2 \Vert f_{0;t} \Vert ^2 / 2} \, \mathrm{e}^{C N \lambda ^3 {\left| \left| \left| O \right| \right| \right| }^3 \exp (C (1+ \Vert v \Vert _1 + \Vert v \Vert _\infty ) t)} \; . \end{aligned}$$

Therefore,

$$\begin{aligned}&\frac{1}{N} \log {\mathbb {E}}_{\psi _{N,t}} \, \mathrm{e}^{\lambda \left[ \sum _{j=1}^N (O^{(j)} - \langle \varphi _t , O \varphi _t \rangle ) \right] } \\&\quad = \frac{1}{N} \log \, \left\langle \Omega , {\mathcal {W}}^*_N (t;0) \mathrm{e}^{\lambda \mathrm{d}\Gamma (q_t \widetilde{O}_t q_t) + \lambda \sqrt{N} \phi _+ (q_t O \varphi _t )} {\mathcal {W}}_N (t;0) \Omega \right\rangle \\&\quad \le \frac{\lambda ^2}{2} \Vert f_{0;t} \Vert ^2 + C \lambda ^3 {\left| \left| \left| O \right| \right| \right| }^3 \exp (C (1+ \Vert v \Vert _1 + \Vert v \Vert _\infty ) t) \end{aligned}$$

for all $$\lambda \le {\left| \left| \left| O \right| \right| \right| }^{-1} \mathrm{e}^{-C ( \Vert v \Vert _\infty + \Vert v \Vert _1) t}$$.

$$\square $$
